# Variability analysis of LC-MS experimental factors and their impact on machine learning

**DOI:** 10.1093/gigascience/giad096

**Published:** 2023-11-20

**Authors:** Tobias Greisager Rehfeldt, Konrad Krawczyk, Simon Gregersen Echers, Paolo Marcatili, Pawel Palczynski, Richard Röttger, Veit Schwämmle

**Affiliations:** Department of Mathematics and Computer Science, University of Southern Denmark, 5230 Odense, Denmark; Department of Mathematics and Computer Science, University of Southern Denmark, 5230 Odense, Denmark; Department of Chemistry and Bioscience, Aalborg University, 9220 Aalborg, Denmark; Department of Health Technology, Technical University of Denmark, 2800 Kongens Lyngby, Denmark; Department of Biochemistry and Molecular Biology, University of Southern Denmark, 5230 Odense, Denmark; Department of Mathematics and Computer Science, University of Southern Denmark, 5230 Odense, Denmark; Department of Biochemistry and Molecular Biology, University of Southern Denmark, 5230 Odense, Denmark

**Keywords:** machine learning, deep learning, data mining, statistics, bioinformatics, proteomics, mass spectrometry, transfer learning

## Abstract

**Background:**

Machine learning (ML) technologies, especially deep learning (DL), have gained increasing attention in predictive mass spectrometry (MS) for enhancing the data-processing pipeline from raw data analysis to end-user predictions and rescoring. ML models need large-scale datasets for training and repurposing, which can be obtained from a range of public data repositories. However, applying ML to public MS datasets on larger scales is challenging, as they vary widely in terms of data acquisition methods, biological systems, and experimental designs.

**Results:**

We aim to facilitate ML efforts in MS data by conducting a systematic analysis of the potential sources of variability in public MS repositories. We also examine how these factors affect ML performance and perform a comprehensive transfer learning to evaluate the benefits of current best practice methods in the field for transfer learning.

**Conclusions:**

Our findings show significantly higher levels of homogeneity within a project than between projects, which indicates that it is important to construct datasets most closely resembling future test cases, as transferability is severely limited for unseen datasets. We also found that transfer learning, although it did increase model performance, did not increase model performance compared to a non-pretrained model.

## Background

**Figure 1:**
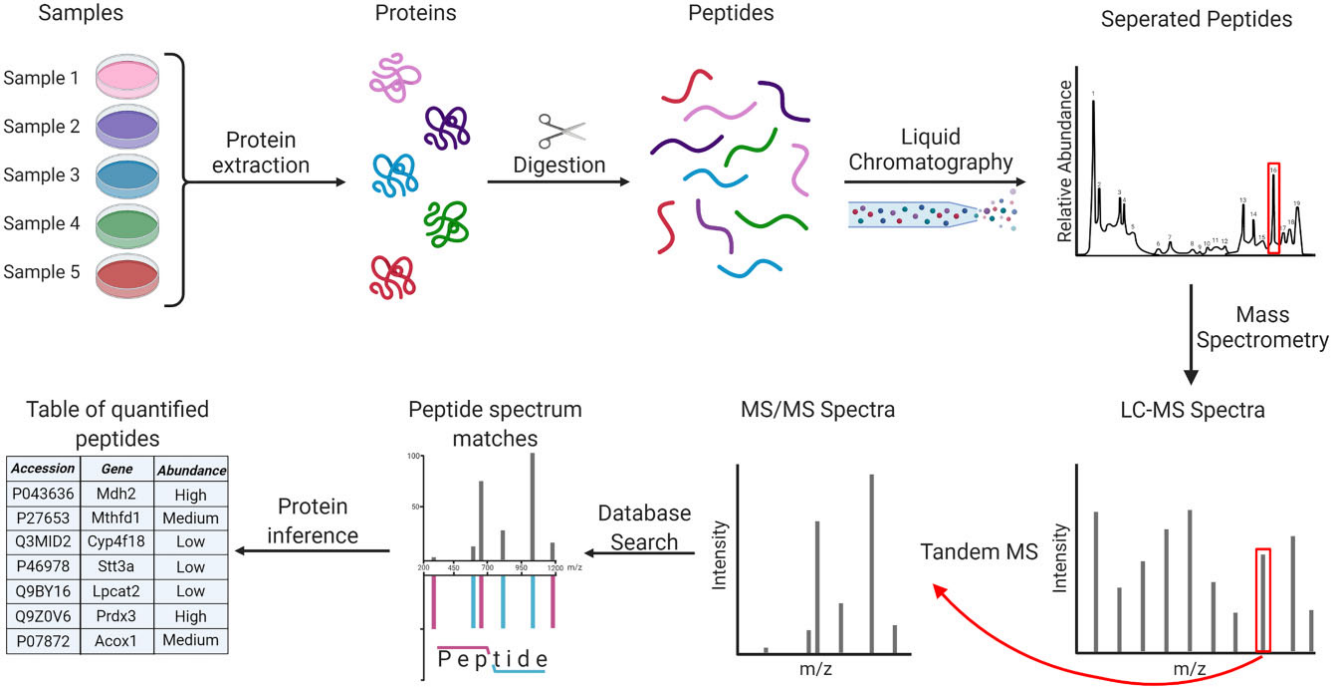
Simplified workflow of a mass spectrometry–based proteomics experiment. First, proteins are extracted from the biological samples, after which they are digested into peptides using enzymes, most often trypsin. Next, peptides are chromatographically separated and injected into the mass spectrometer, where they are measured according to the mass over charge (*m/z*) and abundance (MS1). MS1 spectra from all peptide precursor ions are reported, and certain peptides are chosen for tandem mass spectrometry (MS2), where they are fragmented along their amino acid backbone and identified by having their MS2 spectrum matched to a database of theoretical spectra. Lastly, peptides are quantified and summarized into proteins [14–16]. Created with BioRender.com.

Large-scale studies of proteomes are essential to our understanding of the biological processes within an organism. The leading technology for characterizing thousands of proteins is liquid chromatography–mass spectrometry (LC-MS), which enables high-throughput quantification of protein abundances in a biological sample [1, 2] (Fig. 1).

LC-MS has become the standard within proteomics procedures and continues to generate vast amounts of data, which, due to increasing demands from journals and reviewers, are often made publicly available in data repositories. This change has led to numerous public datasets being registered in online repositories such as the ProteomeXchange (PX) consortium [3]. The PXC contains references to over 17,000 projects, and its largest member, PRIDE, has more than a million raw files. Each raw file contains an average of 6.778 MS1 and 32.016 MS2 spectra, which amounts to over 39 billion mass spectra. These data repositories provide an invaluable resource for data repurposing to address novel biological questions or to benchmark new computational techniques for proteomics data analysis.

While efforts in harmonizing data accessibility within PX and standardizing the computational pipelines are ongoing [3], repurposing data from these repositories comes with a significant entry barrier, as they do not yet have any systematic criteria for metadata or data types.

Due to the advancements in machine learning (ML) model development, there is now an increasing interest in repurposing these rich LC-MS data to train complex ML models that can produce new insights and results not achievable by previous computational methods [4]. However, the large diversity of experimental procedures and biological systems requires careful consideration when applying bioinformatics methods to larger extracts of publicly available data, as ML relies on careful balancing to reach optimal and correct performance.

Multiple ML algorithms and methods have been applied to MS data, such as regression models [5], random forest [6], and, more recently, neural networks [7]. Machine learning applications in proteomics are primarily focused on 2 aspects: (i) improving current methodologies such as database searches or de novo sequences or (ii) predicting physicochemical peptide properties such as LC-MS/MS spectra, retention time, or posttranslational modifications (PTMs) [8–10]. Deep learning (DL) approaches function by generalizing the data, thereby generating distributions of the training data. However, due to the high complexity and large noise found in LC-MS data, many of the current approaches suffer from limited transferability, as they utilize synthetic, limited, or heavily stratified datasets for the purpose of training and testing their models [8, 11, 12]. These issues are further exacerbated by technical advances in the field, such as ion mobility [13], which further increases the complexity and diversity of the data. The majority of current ML methods within computational proteomics also rely on unique and complex postprocessing pipelines, such as peptide-specific indexed retention time (iRT) calculations, rendering the methods difficult to replicate and reducing their application range outside the original publication.

One of the biggest shortcomings in machine learning, particularly deep learning, is the problem of under- and overfitting. These refer to situations in which a model performs too well on the training data (overfitting) and generalizes poorly on unseen test data or not well enough on the training data (underfitting) and subsequently also on unseen test data. Despite multiple attempts and the breadth of available data, these problems are still present in the field of predictive proteomics.

In this article, we investigate the general reusability of public mass spectrometry data for machine learning applications, specifically focusing on potential pitfalls that could result in poor translatability to independently sampled data sets. We will do so by performing statistical analyses on the effect of the experimental setups on the variability of the generated data and see how these effects impact the predictive capabilities of state-of-the-art deep learning models. This work is expected to have an impact on the data selection process in predictive proteomics, elevating the capabilities of current and future models, as well as highlighting the necessity for appropriate preprocessing and algorithmic choices.

### Data description

For a comprehensive representation of publicly available MS data, we analyzed data from ∼60,500 raw files across ∼820 PRIDE projects, totaling ∼60 TB of raw files and metadata. In total, 546 projects containing 33.426 raw files were used for neural network testing. All selected data had been previously analyzed with MaxQuant [17].

**Figure 2:**
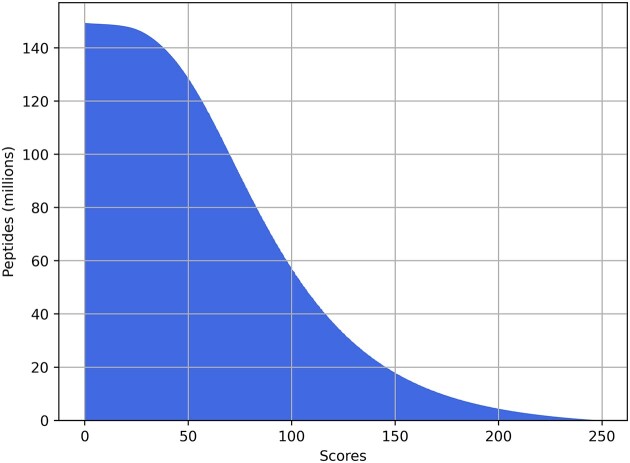
Andromeda score (MaxQuant) distribution plot of the ∼151 M unmodified peptides in the complete data.

The full dataset was gathered from randomly sampled projects on PRIDE using MS2AI [18]. We restricted the retrieval to data from standard bottom-up proteomics experiments. We also did not have any initial queries on experimental or sample preparation, resulting in data from a wide breadth of sources from which we have subsampled smaller datasets for in-depth analyses (Table 1). In total, we gathered spectra and metadata for ∼151 M individual unmodified peptides (Fig. 2).

**Table 1:** Overview of datasets used in model training or testing. All subset datasets are randomly sampled from the full ∼151 M peptide dataset, with Andromeda score and subset filters described in the “Minimal score” and “Description” columns, respectively. No other filters were added when sampling. We also annotated the size of the subset dataset in the “Peptide counts” column, with a 2 M peptide count limit for each dataset.

Name	Section	Minimal score	Peptide counts	Description
*PT17*	1	100	750,000	Dataset constructed from PXD004732
*PT19*	1	100	750,000	Dataset constructed from PXD010595
*Limit*	1	100	750,000	750,000 peptides, excluding PXD004732 and PXD010595
*Wide*	1	150	2,000,000	2,000,000 peptides, excluding PXD004732 and PXD010595
*Long*	2	150	2,000,000	Gradient length equal to or above 100 minutes
*Short*	2	150	2,000,000	Gradient length equal to or below 60 minutes
*Lower*	3	100	125,000	Peptides with *m/z* values below 360
*Upper*	3	100	125,000	Peptides with *m/z* values above 1,300
*Human*	Supp.	150	2,000.000	“Human (*Homo sapiens*)” from PRIDE metadata
*Mouse*	Supp.	150	2,000,000	“*Mus musculus* (mouse)” in PRIDE metadata

## Results and Discussion

We performed a thorough statistical assessment of the data and trained multiple neural networks in order to gauge the variability and evaluate the transferable capabilities of state-of-the-art models. However, in the field of predictive proteomics, especially in retention time prediction, it is common to apply transfer learning to pretrained models. This is done as a result of the poor transferability of the original networks, as the models do not achieve setup independence by being constrained to the experimental settings of the training data. However, transfer learning requires a large amount of data in a format suitable for machine learning, as well as significant computational expense, making it both data and computationally intensive. Additionally, it also requires expertise in both machine learning and programming. To test the effectiveness of transfer learning in predictive proteomics, we exhaustively transferred all of the trained models to every other dataset in the same section to assess the impact of transfer learning on performance.

All models were measured with several metrics—namely, retention time error (RTΔ), mean squared error (MSE), and mean absolute error (MAE)—and the performance for all metrics can be found in the supplementary evaluation_metric_report.pdf file. However, when analyzing the model performances, we observe near-identical metric ratios between training, validation, and testing across various evaluation metrics. This suggests that the outcomes remain invariant across metrics and do not alter the conclusions drawn from the models’ performance. For this reason, we have decided only to report the RTΔ values and refer to the evaluation metric report for further metric comparison. RTΔ measures the average time difference between predicted RT values and actual values and is the proprietary metric used by the DLOmix package. DLOmix is a software integration of popular DL models used in proteomics, which we used to construct the Prosit model.

In this article, we use the transferability of model predictions as a measure of data variability by training models to predict the elution times of identified peptides based on a range of measurable factors. While we try to stratify datasets to keep certain parameters constant, there is a range of underreported and unextractable information that we are incapable of controlling, which is often introduced from the sample preparation or the LC system.

During sample preparation, the efficiency of protein digestion can cause incomplete digestion or nonspecific cleavage, which can result in the presence of partially digested peptides. These altered peptide species can significantly influence the elution behavior, adding complexity and variability to the analysis. Additionally, sample cleanup protocols are employed to remove interfering impurities like salts and detergents, which, based on the efficiency of the procedure, may leave behind residual impurities that can affect the peptide elution process. Moreover, the sample matrix composition introduces the matrix effect, which can impact peptide ionization and detection.

In the LC system, column chemistry determines the interactions between peptides and the stationary phase, while column dimensions affect separation efficiency and resolution. Modifying either the mobile or stationary phase composition can alter the peptide's polarity, ionic strength, binding affinity, and so on, altering the elution time and profile. The use of traps or precolumns in the LC system introduces variability by selectively retaining peptides or interfering with separation. LC temperature influences molecular interactions, including solvent viscosity, peptide conformation, and peptide–solvent interactions, thereby affecting peptide elution and separation.

These are just some of the unextractable factors that can contribute to the variability and affect the elution times of peptides. Unfortunately, we are unable to measure or control these factors. However, by having a sufficiently large dataset and randomly drawing data points from a range of thousands of raw files, we aim to mitigate their impact on the model predictions and conclusions, also by assuming that the majority is based on standard LC setups.

### Single- vs. multiproject variability

**Figure 3:**
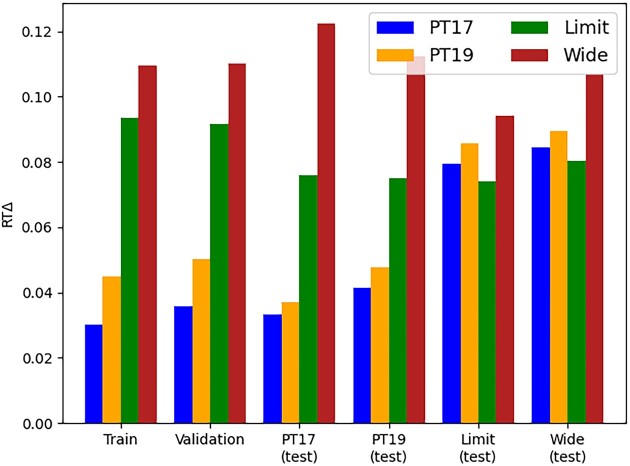
General variability model performance comparison. Each model was trained and validated on its original source datasets and then cross-tested for all test datasets. We compared the training and validation for all models, as well as cross-testing datasets and their respective model performance in terms of RTΔ.

In our model comparisons, we found that the models trained on the *PT17* and *PT19* datasets performed significantly better than those models trained on the *Limit* and *Wide* datasets during both training and validation (Fig. 3). Interestingly, the *PT* models also outperformed the *Wide* model and performed comparably to the *Limit* model when testing on the wide and limited datasets.

While we expected the training and validation of the *PT* models to outperform the *Wide* and *Limit* model, we did not expect the *PT* models to outperform the *Wide* and *Limit* models on the *Wide* and *Limit* test datasets. Furthermore, we also found that the *Limit* model outperformed the *Wide* model for all test cases, suggesting that increasing the amount of data and using stricter scoring criteria does not necessarily improve the performance of trained models and may even cause the models to underfit. One possible explanation for this is that longer and more complex peptides, which are easier to detect and generally receive higher peptide identification scores, also exhibit more variability in their elution times. We tested this and found that the peptides in both *PT* datasets have average peptide lengths of ∼12, with the *Limit* dataset containing a longer average peptide length of ∼13 and the *Wide* dataset containing an even longer average length of ∼14.

The reduced performance observed in the randomly sampled datasets is also possible due to the presence of multiple variability-inducing factors in the experimental setup. These factors, such as the type of MS instrument or the selected species, are often difficult or impossible to account for when relying on large bulks of public data. Changing these could result in considerably different model performances (Supplementary Fig. S1). In contrast, the *PT* datasets were measured under the same conditions on synthetic peptides, which reduces the presence of such variability-inducing factors. Additionally, the *PT* datasets have the advantage of using one single gradient length, while the limited and wide datasets use multiple different gradient lengths.

**Figure 4:**
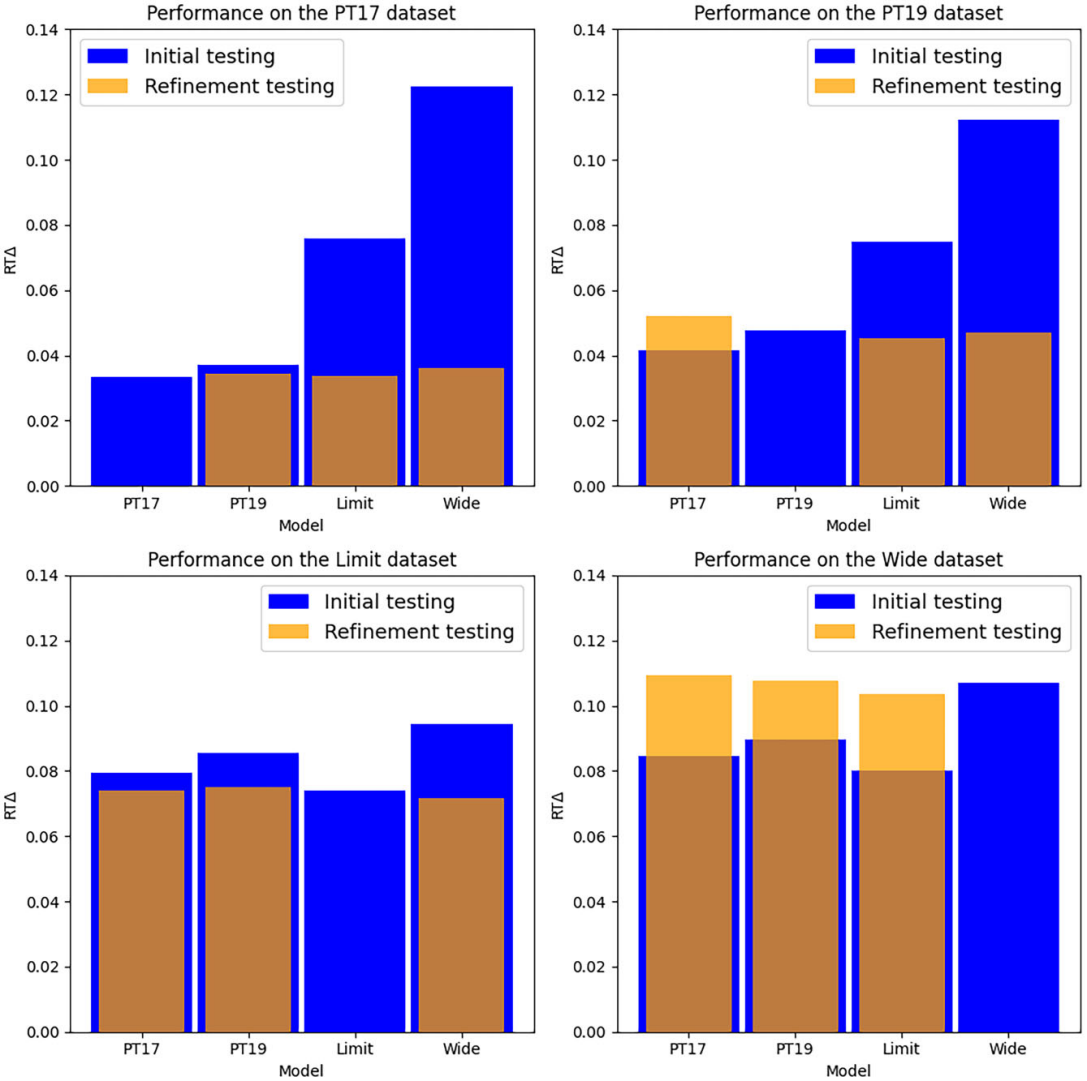
General variability transfer model performance comparison. Each model was transferred to each of the external datasets and retested on all 4 datasets. Datasets are separated by plots, denoting the performance difference of each model when trained on or transferred to identical datasets. Each bar has the original test metric in blue, and the transfer learning test metric is overlaid in orange.

In order to evaluate their transferability, we applied transfer learning to all 4 models and found that, while some of the models improved performance compared to previous external testing, they mostly performed similarly to nontransferred models on the same dataset (Fig. 4). In the case of the *Wide* dataset, transfer learning actually resulted in reduced performance for all transferred models. This suggests that the *Wide* dataset had significantly higher levels of heterogeneity between training and testing data compared to the other datasets. While these results show that transfer learning can be beneficial in certain scenarios, they also showed that most of the models simply improved or regressed to the transfer dataset. However, even if transfer learning did not provide significant predictive benefits, it did reduce the time needed to train the models by an average of 5.5% by converging faster (Supplementary Table S2).

### Gradient lengths

The interactions between the peptide and the stationary and mobile phases of the liquid chromatography system determine the retention time of a peptide in an LC-MS/MS system. In identical setups, the retention time of a peptide is considered reproducible [5].

**Figure 5:**
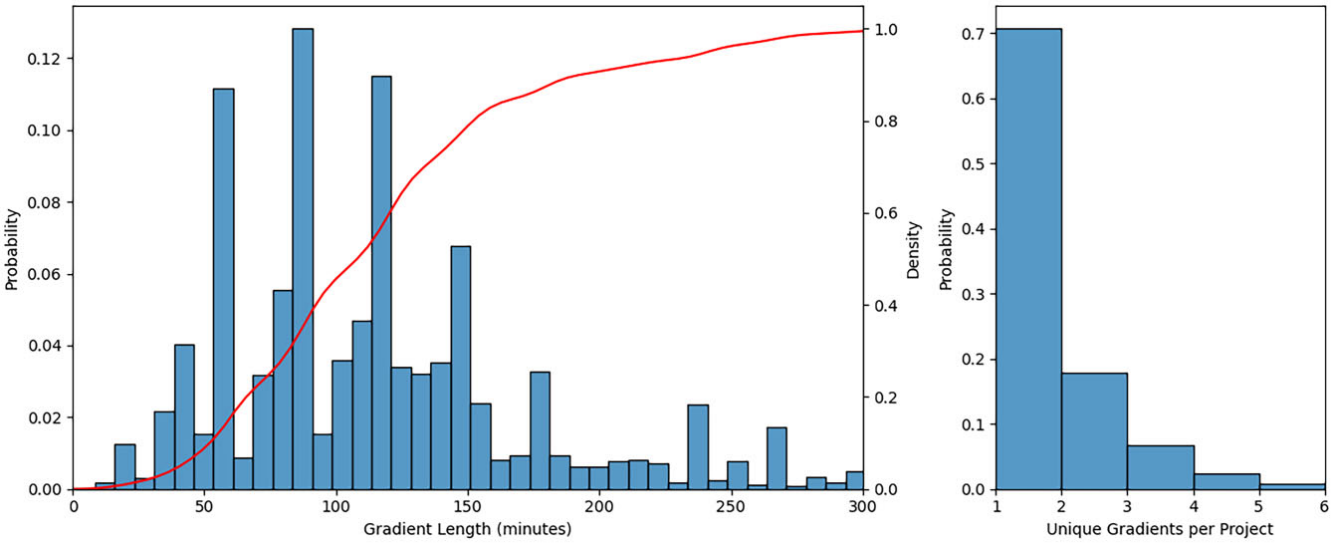
Gradient length distribution and unique gradient per project. We illustrated the gradient distribution of all 60,000+ raw files overlaid with the cumulative distribution (A). Also illustrated is the number of unique gradients found within any of the 820 projects (B).

Plotting the distribution of gradient length for all raw files with an overlaid cumulative distribution function (Fig. 5A), we observe significant peaks at 60, 90, and 120 minutes, with ∼60% of all gradients being 0–120 minutes in length and the longest gradient being 800 minutes. While we did find single projects with as many as 15 different gradients, we also found that 70% of the 820 projects kept the same gradient length for all files, while only ∼5% employed more than 2 unique gradients (Fig. 5B), indicating high levels of consistency in instrument configurations within individual projects.

The results of our deep learning use case showed that the *Short* gradient model performed significantly better than the *Long* gradient model (Supplementary Fig. S2). This is further supported by its decreased performance on the *Long* gradient test dataset compared to the *Short* model.

These findings suggest that peptides from longer gradients generally express higher variability compared to peptides from shorter gradients, even after attempted peptide normalization. It also indicates that our normalization method for the effective gradient, which aims to mimic the linear iRT calculations used in the original Prosit study, may not be effective for all gradients and raw files, reiterating the necessity of targeted postprocessing pipelines.

**Figure 6:**
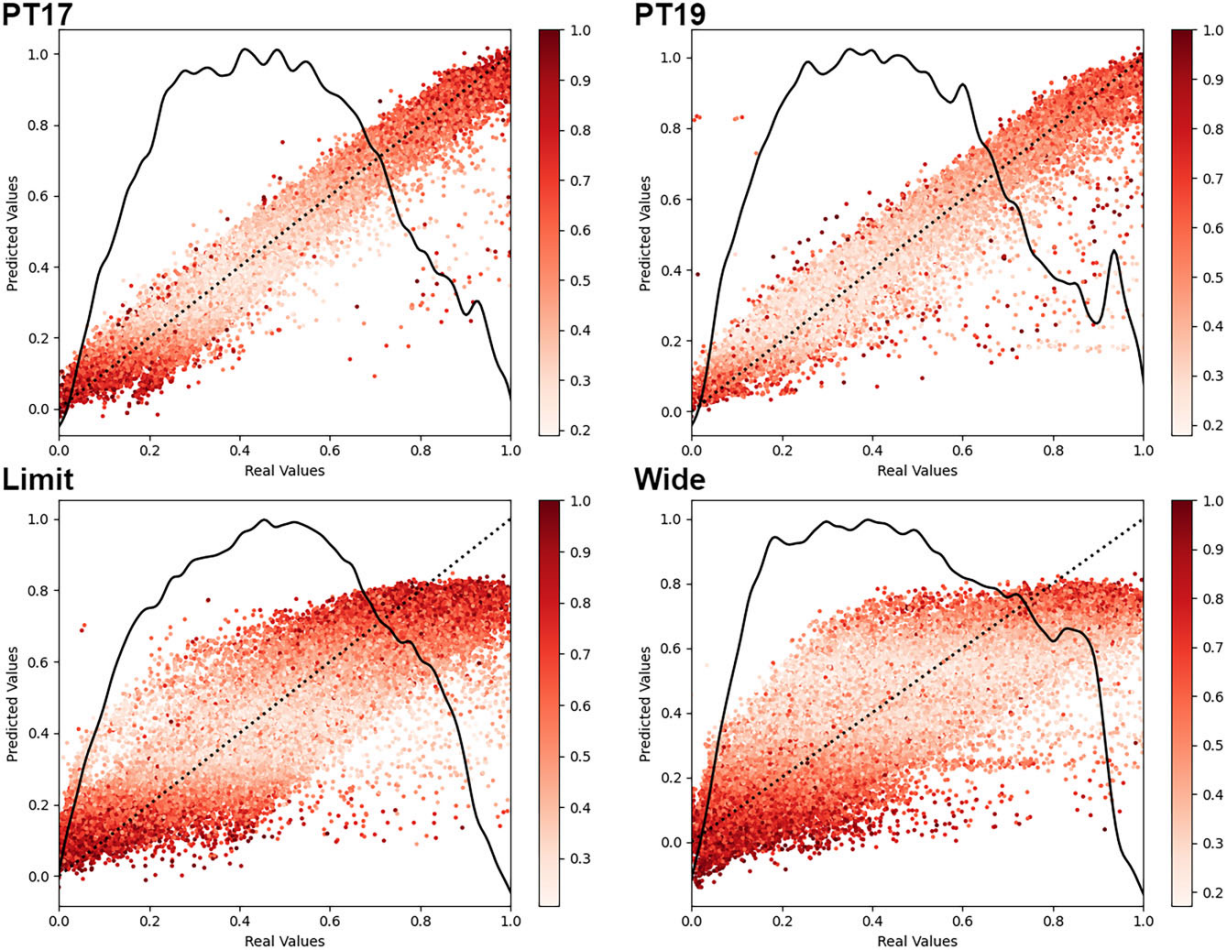
Bayesian model uncertainty estimates of single- vs. multiproject variability models. For each of the models, we conducted model inference 25 times with layer dropouts applied at original rates. Each data point is an average of the inference results, with the colorization of the dots indicating the normalized relative variability of each peptide. For each of the datasets, we overlaid a distribution plot of the dataset retention time values as a solid line and a linearity assessment (y = x) to facilitate a comparison of the model predictions with perfect alignment as a dotted line.

Performing inference dropout on all models in the previous section shows that all of the models exhibit significantly higher uncertainty for the earliest and latest eluted peptides compared to those eluted closer to the middle of the gradient (Fig. 6). Additionally, we observe that the *PT* models show a more linear prediction gradient than the *Limit* and *Wide* models, further suggesting the controlled nature of the ProteomeTools dataset output peptides in a more linear gradient, which fits better for our first–last peptide gradient normalization. We also observe less overall uncertainty in the PT models, likely because they were trained on datasets with fewer peptides, lower average peptide retention times of approximately 32 minutes, and unified gradients, whereas the average retention times of the limit and wide datasets were significantly higher at 60+ minutes from multiple gradient lengths. This suggests that longer gradients lead to an increase in data variability and model uncertainty.

Performing transfer learning on the gradient length models (Supplementary Fig. S3), we observe that refining the *Long* model to the *Short* dataset resulted in a significant performance improvement. However, refining the Short model to the Long dataset did not result in any significant change in performance, although it still outperformed its nontransferred counterpart. Unlike what we observed in the previous section, transfer learning of the gradient length models came at an increase or a stagnation in performance, indicating that the models retained information from the initial training datasets. We also note that transferred models took, on average, 25% longer to train compared to nontransferred models (Supplementary Table S3).

### Mass-to-charge range filter

MS instruments have a range of setup parameters that can tailor the experiment to the needs of researchers. The *m/z* range filter is one of those parameters, as it restricts peptide data acquisition to a given *m/z* range. However, for data repurposing, this range can also lead to a biased dataset for machine learning, as the sample might have contained a large range of peptides not reported by the instrument. While the filter settings do exclude certain data points, it should be noted that most database searches also have cutoffs for shorter peptides due to noise at the lower end of *m/z*, making peptide identifications in this space more unlikely even when peptides are present.

**Figure 7:**
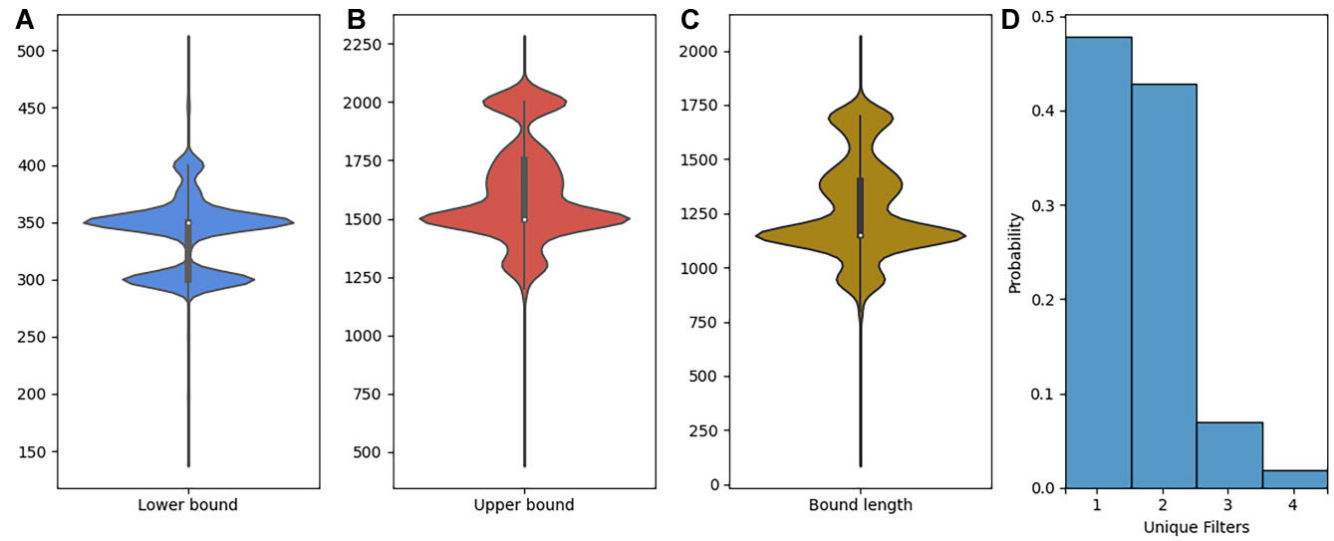
Illustration of the variability of *m/z* range filters found in different projects. Violin plots depicting the *m/z* filter bound distributions, with the lower bound plotted in blue (A), upper bounds plotted in red (B), filter length plotted in yellow (C), and a histogram of the total number of unique filters found within projects (D).

When plotting the *m/z* filters of the mass acquisition range (Fig. 7), we observe significantly more variability in the upper bound compared to the lower bound, meaning that our upper bound is highly correlated to the length of the filter. All violin plots exhibit a peak at 1 specific value, 350 for the lower bound and 1,500 for the upper bound, with a corresponding peak at 1,150 for the filter lengths. These peaks correspond to the most commonly used filter, which accounts for 33.9% of all raw files.

We also observed that 47% of all projects applied a single filter across all raw files, 43% of projects applied 2 filters, and only 10% of the projects applied more than 2 unique filters. Consequently, there is significant homogeneity within a project, while between projects, the filters can differ considerably. If datasets are constructed using only 1 or a few unique filters, large portions of the data space may never be used for training, potentially limiting the transferability of the models (Supplementary Fig. S4).

**Figure 8:**
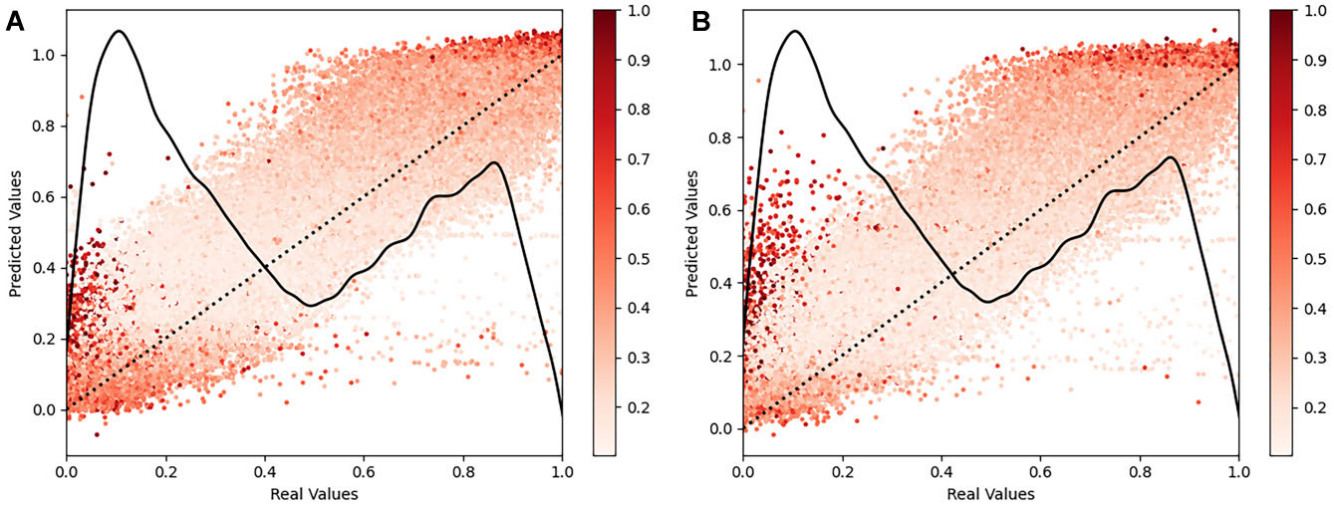
Bayesian model uncertainty estimates for out-of-bounds peptides. For *PT17* (A) and *PT19* (B), we conducted model inference 25 times with layer dropouts applied at original rates. Each data point is an average of the inference results, with the colorization of the dots indicating the normalized relative variability of each peptide. For each of the datasets, we overlaid a distribution plot of the dataset retention time values as a solid line and a linearity assessment (y = x) to facilitate a comparison of the model predictions with perfect alignment as a dotted line.

We also tested the model performance of the *PT17* and *PT19* models on peptide datasets only containing peptides outside of the original filter bounds (out-of-bounds [OOB], Supplementary Fig. S5) and found that model performance was significantly worse when compared to the source test datasets. The OOB testing performance is also significantly worse when compared to *Limit* and *Wide* testing (see Results and Discussion), which also contained peptides in the OOB range. Interestingly, the models performed slightly worse on heavier OOB peptides than on lighter OOB peptides (Fig. 8), despite lighter peptides exhibiting higher individual variability and the distribution of lighter peptides being more concentrated at one end of the distribution compared to the heavier peptides (Supplementary Fig. S6). Similarly to Fig. 6, we observe significantly more model uncertainty tied to earlier and later eluted peptides.

The significant reduction in performance we observed in the OOB testing suggests that the models do not learn the underlying nature of amino acid (AA) weights, folding, and physicochemical properties, factors that impact RT as well as the ionization and detectability of peptides, as much as they memorize the retention times of certain peptide sequence patterns [12].

### Fragmentation patterns

We also investigated the variability and content of fragmentation spectra in public data. In this case, as fragmentation does not influence peptide retention time, we will only consider the theoretical impact on deep learning applications like fragmentation pattern predictions and rescoring.

A perfect peptide fragmentation spectrum is a theoretical concept that consists of a discrete set of all characteristic peaks defined only by the peptide sequence. In reality, fragmentation spectra only contain subsets of these theoretical peaks with patterns based on the background contaminants from the instrument, fragmentation technique, collision energy, and more. In order to understand the challenges and limitations of the MS2 spectra for machine learning algorithms, we analyzed MS2 spectra peaks from 86 randomly sampled raw files containing more than 768 million peaks, allowing us to visualize the peak distributions found in MS2 spectra.

**Figure 9:**
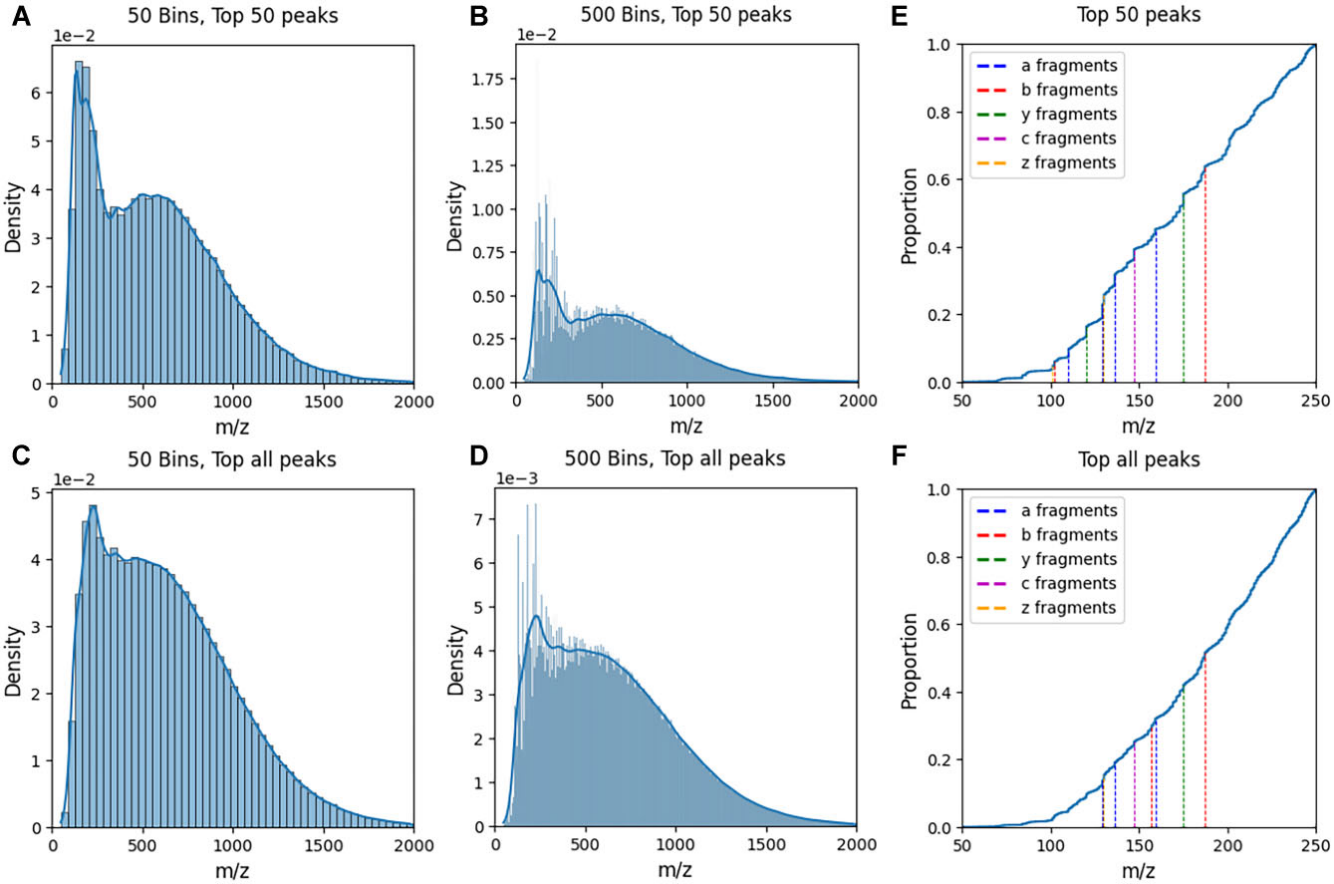
Illustration of how peak picking and peak binning affect the MS2 peak density plot and single amino acid density. The density distributions of peak *m/z* values shown were obtained without peak picking (C, D) and with the top 50 peaks (A, B). The spectra were then imposed into 50 (A, C) and 500 (B, D) bins. E,F: for each value of peak picking, we also illustrated the cumulative distribution plot of the peaks in 50 to 250 *m/z* with single AA residues overlaid. No stratification on the fragmentation method or its settings has been considered during this analysis.

When looking at the distribution of all *m/z* values found in the 86 randomly sampled files, we observe a clear bimodal distribution independent of peak selection or bin sizes; 1 distribution is located at ∼50 to 250 *m/z* and the second distribution at ∼250 to 2,000 *m/z*. The distribution at the lower *m/z* range disrupts the expected normally distributed peptide fragment ions found at 250 to 2,000 *m/z* and consists of clearly distinguishable high-density peaks corresponding almost exclusively to single amino acid residues (Fig. 9E, F) and some background noise. The cumulative distribution plots in Fig. 9 show the singly charged amino acid residues, where amino acids are annotated if the *m/z* of the peak matches the collision ions *a, b*, or *y* or the electron-transfer ions *c* or *z*. The most abundant amino acid peaks are highlighted in Supplementary Table S1, and we observe that these peaks become more frequent at higher levels of peak selection (Supplementary Figs. S7–S8). It should be noted that the exact AA contribution to some peaks is uncertain, as multiple AA ions match the same *m/z* peaks.

One thing not taken into consideration in this analysis is the method and energies used during fragmentation. The fragmentation technique, such as collision-induced dissociation (CID), high-energy C-trap dissociation (HCD), or electron-transfer dissociation (ETD), has a significant impact in determining the patterns of peptide fragmentations. This is due to each technique applying varying amounts and types of energy to the precursor ions, leading to distinct bond cleavage pathways and producing specific fragment ions [8]. Along with the fragmentation technique, it is also essential to discuss the fragmentation energy, often called normalized collision energy (NCE) for CID and HCD, and charge state-dependent reaction time for ETD. The stability of the created fragment ions depends primarily on the size, polarity, and charge on either side of the cleavage, and the energy with which the fragmentation occurs determines which bonds can be broken [19, 20]. This also means that higher energy fragmentations are more likely to create single AA ions [21, 22]. Utilizing the fragmentation-specific ion patterns, either by inclusion or stratification, has already been proven successful for both top-down and bottom-up predictive proteomics [7, 8, 23, 24]. Due to the complexity of LC-MS/MS fragmentation patterns across fragmentation techniques and energy, some projects utilize project-specific peptide libraries to increase the identification of desired peptides. Even though the usage of peptide libraries will not directly affect the variability of peptide patterns in LC-MS or LC-MS/MS, they can still affect identified variability by having a significantly higher probability of identifying peptides in the library while not identifying a large number of otherwise present peptides. Recent developments in DL methods have enabled enhanced or library-free peptide identifications, which, with future improvements, could increase efficacy without biasing the identification [25, 26].

## Conclusions

Mass spectrometry remains a powerful tool to quantify thousands of protein abundances in biological samples. Analysis of the raw experimental data is increasingly dependent on suitable computational methods [27], with a major focus on algorithms for peptide identifications and protein quantifications. However, despite a variety of different statistical, conceptual, and graph approaches, methods such as database search engines still suffer from limitations in both accuracy and runtime. Novel machine learning methods hold the promise of advancing the analysis of upcoming data, as well as having a high potential for repurposing the ample body of public data for the retrieval of valuable new biological information.

In this article, we have investigated and highlighted some of the main sources of variability found within the high-throughput MS data. We identified a range of factors that increase variability in the data-generating process and analyzed the homogeneity of the variability within a project when comparing different projects. Our main finding from the statistical analyses was that global variability, which is found between projects, is significantly larger than internal variability, which is found between files in the same projects. This is exhibited through instrument settings, sample preparation, and experimental choices, all of which are significantly more homogeneous within any given project compared between projects. Furthermore, we also wanted to see how these sources of variability impacted ML capabilities by training Prosit retention time predictors on each source individually, whenever applicable.

We trained 9 Prosit models, tested these models on 27 datasets, and performed transfer learning 14 times. An alternative approach would have involved performing multiple randomized data splits for each model and averaging the results to provide a more comprehensive assessment of model performance. However, we chose not to pursue this option due to their computational costs. Our findings show that training models on data most closely resembling real-life test cases are crucial, as the models' ability to generalize outside the training data confinements was severely limited. This was illustrated by the *PT* models outperforming any other model during training and validation while having considerable performance drops when tested on randomly acquired data or peptides not in the original *m/z* range.

Our results also found evidence that transfer learning can occasionally improve the performance of a pretrained model. However, the most common scenario we observed was that models ended up mimicking nontransferred models for the same dataset while not reducing the average amount of epochs needed for convergence. This tendency resulted in model regression in 5 of the 14 cases and only resulted in model improvement in 1 of 14 cases. While our findings do indicate that models need to be trained on datasets from representative sources, they do not indicate that transfer learning outperforms training a new model in terms of accuracy or computational needs (Supplementary Tables S4 and S5).

Since a representative dataset is needed, we argue that a research environment either has to train specialized models to their data collection methods or generate an unbiased dataset from publicly available data sources that attempt to mimic the intended posttraining application through software such as MS2AI [18].

We further found that fragmentation spectra are rich in yet neglected information. Given the abundant single-residue fragment ions, particularly at higher activation energies, considerable amounts of internal ions should be present. This information has been so far mostly untapped due to the complexity of including internal ions in database search and spectrum prediction. Advanced machine learning methods might be capable of making sense of these ions despite their noisy and ubiquitous nature.

We note that a prevailing issue with the current data repositories is the missing or mislabeling of metadata. With the ongoing standardization efforts in large repositories such as PRIDE [28], this issue should fade over time. Through the analysis, we also identified the need to report more details about the experimental design, the data acquisition, and the postprocessing in a comprehensive and standardized way to make them amenable as additional input for machine learning applications and thus allow for the direct training of the confounding factors.

## Methods

We use different methods to evaluate the variability caused by different setup parameters of the LC-MS experiments and their effect on ML transferability to unseen data. To assess the impact of biases and experimental heterogeneity, we trained identical deep learning models over a range of data properties and compared their results. We used the Prosit retention time model with peptide sequence and retention time as input and output, respectively. The Prosit model architecture consists of a sequence embedding layer, a bidirectional GRU layer, and an attention layer, followed by fully connected dense layers. The retention time of all peptides in a raw file has been linearly normalized to an effective gradient, spanning between the first (0) and last (1) identified peptide to mimic the iRT calculations performed in the original study. No further data refinement or reannotation has been applied to the files. Initially, we followed the hyperparameter setup described in the Prosit study but found that 32 epochs were not sufficient for model training convergence. As a result, we increased the training to 100 epochs and applied a 20-epoch patience for early stopping instead. All other parameters were identical to those described in the original study [8]. The Prosit deep learning architecture was implemented by using the DLOmix framework [29], and all modified peptides were removed due to DLOmix constraints.

For all trained networks, we sampled 10% of each dataset as a hold-out test set on which all testing was conducted. The remaining data was split into training and validation sets with a ratio of 80:20. For datasets composed of multiple PRIDE (RRID:SCR_003411) projects, the hold-out datasets consist of separate projects that were randomly sampled, whereas for datasets consisting of a single project, the hold-out dataset consists of randomly sampled raw files. This provided the most accurate heterogeneous test scenarios without overlap across projects or MS runs. Furthermore, the training and validation datasets were split by peptide sequences, meaning that no peptide will be present in either the training or the validation datasets. However, since the testing data were randomly split at the project or file level, these may contain sequences that are also present in the training datasets.

The data acquisition, filtering, model training, and testing were managed using MS2AI with MongoDB (RRID:SCR_021224) in Python 3.8 with an NVIDIA v100 GPU. The data were acquired in November 2021 with the extractor API with the options “-p -mo -t 128,” which allows for en masse data acquisition from PRIDE (-p) while only fetching MaxQuant (RRID:SCR_014485) information (-mo) and increasing thread counts to 128 to allow for faster runtime (-t 128). This requires the current version of the PRIDE metadata, which is downloaded using the scraper API and the -db option. The filtering was performed using the filter API using the -q option with MongoDB or string filters available in the GitHub repository. The model training and testing were performed using the network API with “-t prosit -e 100 -sos -s *n*” to train a Prosit model for 100 epochs, with training and validation being split based on unique sequences and a given seed for consistent training, validation, and test splitting. When performing transfer learning, the only difference is “-t prosit-ID,” which uses the weights of the trained model with the same ID. Model training times varied from 4 to 10 hours based on the dataset size and epochs needed to converge. All code and seeds for the runs are available in the GitHub repository.

We utilized a Bayesian approximation of the model uncertainty by performing model inference with dropout enabled [30, 31]. The real retention time values are then plotted against the mean predicted values, with the color of the data point corresponding to the normalized variances of the predicted values. The dropout for inference testing was applied to all layers where dropout was originally applied, with original dropout rates. This allowed us to not only evaluate the models on their metric performances but also determine the retention time ranges where the models are least certain of their predictions. This method is available in MS2AI network API using the “-id *n*” option to run *n* dropout tests and automatically generate the data visualization plots.

### Single- vs. multiproject variability

In order to measure the difference in variability not caused by individual factors but instead caused by systemic changes in experimental protocol, we compared the model performance of 2 single-project models to the performance of 2 multiproject models. We did this by training 4 Prosit models on data from 4 different sources: 2017 [32] and 2019 [33] ProteomeTools (RRID:SCR_018535) datasets (*PT17* and *PT19*, respectively) and 2 sets of acquired data from randomly sampled PRIDE projects—one limited to the 750,000 peptides filtered at 100 Andromeda score threshold, which is the score reported by MaxQuant (*Limit*), and one with 2,000,000 peptides filtered at 150 Andromeda score threshold (*Wide*). Alongside the initial training and testing, we also performed transfer learning on all models for all nonsource datasets to compare their initial performance to posttransfer learning performance.

### Spectra and gradient lengths

To compare and analyze the variability in gradient lengths, we extracted the metadata from each raw file (found in the *files.bson.gz*) and the gradient lengths of all runs individually, which we plotted in a histogram against the probability of each gradient length. We then calculated the cumulative distribution function of the gradient lengths for all files, which is overlaid on the histogram. Then, we calculated the number of unique gradients across all files from the same PRIDE accession, allowing us to visualize the variability found within projects when plotting the number of unique gradients in a histogram against the probability of each number of unique gradients.

Along with gradient length visualizations, we also trained 2 Prosit models to evaluate the effect of gradient length on model performance. The models were trained datasets that were divided into 2 groups based on their gradient lengths: short (≤60-minute gradients) and long (>100-minute gradients). The data were randomly sampled from the entire 151 M peptide dataset, and only peptides with ≥150 Andromeda scores were kept. We also performed transfer learning of both gradient models to the opposing datasets. Furthermore, to test model uncertainty across the gradient, we performed model inference with dropout enabled on all 4 models, as described above.

### *m/z* range filter

To visualize and compare *m/z* filters across files and projects, we extracted the *m/z* filter bounds from each raw file and plotted the lower bounds, upper bounds, and the difference between the upper and lower bounds to get the lengths. We then visualized these values in a violin plot in order to see possible patterns or key values in the distributions.

In order to evaluate the impact of *m/z* filters on model performance, we created 2 subsets of data, this time much smaller due to low peptide count: one in which all peptides lie below 360 *m/z* (*lower*) and one in which all peptides lie above >1,300 *m/z* (*Upper*). These bounds were chosen as we are going to reuse the *PT17* and *PT19* models, which have *m/z* filter bounds of 360 to 1,300 m/z, and using these datasets allowed us to evaluate peptides that are outside original filter bounds. As we did not train new models for this section, no transfer learning was applied. Again, we also performed model inference with dropout enabled in order to assess the model uncertainty for these OOB peptides.

### Fragmentation pattern

To analyze the distribution of MS2 peaks, we extracted every peak from 86 raw files [34], where we compared the raw spectra with all peaks preserved to spectra filtered by top *n* peaks based on intensity for 3 values of *n*: 50, 100, and 200. MS2 spectra are often annotated or binned to make them fit into typical ML architectures. To illustrate how this type of binning affects the outcome distribution, we also binned each of the combined peak selected spectra at 50, 100, 200, and 500 total bins from 0 to 2,000 *m/z*. We then calculated the collision ions *a, b*, and *y* as well as the ETD ions *c* and *z* for all amino acids separately. This was done by adding −27, 1, 19, 18, and 2 mass to their single charged molecular residue weights for *a, b, y, c*, and *z* ions, respectively using the web tools "Amino acid residues molecular masses" ( http://www2.riken.jp/BiomolChar/Aminoacidmolecularmasses.htm) and "Proteomics Toolkit" (http://db.systemsbiology.net/proteomicsToolkit/FragIonServlet.html), both accessed 2023, March 20.

## Source Code and Requirements

Project name: MS Review Paper

Project homepage: https://gitlab.com/tjobbertjob/ms-review-paper

Operating system(s): Platform independent

Programming language: Python

Other requirements: Python 3.8, MongoDB

License: e.g., GNU AGPLv3

RRID:SCR_024531.

## Supplementary Material

giad096_Supplemental_File

giad096_GIGA-D-23-00094_Original_Submission

giad096_GIGA-D-23-00094_Revision_1

giad096_GIGA-D-23-00094_Revision_2

giad096_GIGA-D-23-00094_Revision_3

giad096_Response_to_Reviewer_Comments_Original_Submission

giad096_Response_to_Reviewer_Comments_Revision_1

giad096_Response_to_Reviewer_Comments_Revision_2

giad096_Reviewer_1_Report_Original_Submission Juntao Li -- 5/25/2023

giad096_Reviewer_1_Report_Revision_1 Juntao Li -- 8/28/2023

giad096_Reviewer_1_Report_Revision_2 Juntao Li -- 9/24/2023

giad096_Reviewer_2_Report_Original_Submission Luke Carroll -- 6/19/2023

giad096_Reviewer_2_Report_Revision_1 Luke Carroll -- 8/31/2023

## Data Availability

The database files, reference texts, and trained models supporting the results of this article are available in the FigShare repository [34]. The DOME-ML Registry file containing annotations supporting this work is available as a supplementary file attached with this article. Snapshots of our code are archived in Software Heritage, https://archive.softwareheritage.org/browse/origin/directory/?origin_url=https://gitlab.com/tjobbertjob/ms-review-paper.git&snapshot=1246a52f5717c414df138b746e6d54a2712673a1.

## Additional Files

1. DOME-ML Registry File
2. Evaluation metric report
3. Supplementary figures

**Supplementary Fig. S1**. Species model performance comparison. Each model was trained and validated on individual internal datasets and cross-tested on all external datasets as a basis for model comparisons. The x-axis denotes datasets, and the bars denote model performance on the given dataset.

**Supplementary Fig. S2**. Gradient model performance comparison. Each model was trained and validated on individual internal datasets and cross-tested on all external datasets as a basis for model comparisons. The x-axis denotes datasets, and the bars denote model performance on the given dataset.

**Supplementary Fig. S3**. Gradient-based refinement model performance comparison. Each model was refined on all previous external datasets and retested on both datasets. Datasets are separated by plots, denoting the performance difference of each model when trained on, or refined to, identical datasets. Each bar has the original test metric in blue and the refinement test metric overlaid in orange.

**Supplementary Fig. S4**. Illustration of the unseen data space caused by limited search space filters. Illustrated are 3 theoretical search spaces superimposed on top of a theoretical MS1 chromatogram, with unreported peptides at both ends of the data space.

**Supplementary Fig. S5**. Out-of-bounds performance comparison of PT models. The *PT17* and *PT19* model comparison for their individual internal datasets (initial), peptides with *m/z* below 360 (lower), and above 1,300 (upper).

**Supplementary Fig. S6**. Bayesian model uncertainty estimates for out-of-bounds peptides split into 2 datasets. The out-of-bounds datasets are split into 2 datasets: peptides with *m/z* below 360 (left) and above 1,300 (right). Models are separated into rows with *PT17* in the first row (A, B) and *PT19* in the last row (C, D).

**Supplementary Fig. S7**. Density distribution of MS2 peaks with different levels of peak picking imposed at 50 and 100 bins. For each value of peak picking, we also illustrated the cumulative distribution plot of the peaks in 50 to 250 *m/z* with single AA fragmentations overlaid.

**Supplementary Fig. S8**. Density distribution of MS2 peaks with different levels of peak picking imposed at 200 and 500 bins. For each value of peak picking, we also illustrated the cumulative distribution plot of the peaks in 50 to 250 *m/z* with single AA fragmentations overlaid.

**Supplementary Table S1**. Corresponding single amino acid peak table. *m/z*, amino acid, and fragmentation type of MS2 peaks in Fig. 9. “*” does not meet the threshold of 1% in the given *n* and is not annotated on the CDF. Note that 129.1 and 129.2 have been merged to 1 peak, due to rounding error.

**Supplementary Table S2**. Model epochs for training convergence overview of transfer learning models.

**Supplementary Table S3**. Model epochs for training convergence overview of gradient-length models..

**Supplementary Table S4**. Model epochs for training convergence overview of models trained for organisms.

**Supplementary Table S5**. Model epochs for training convergence overview of models trained for instruments.

## Abbreviations

CID: collision-induced dissociation; DL: deep learning; ETciD: electron-transfer and collision-induced dissociation; ETD: electron-transfer dissociation; EThcD: electron-transfer and higher-energy collision dissociation; HCD: high-energy C-trap dissociation; LC-MS or MS1: liquid chromatography–mass spectrometry; LC-MS/MS, MS/MS, or MS2: tandem mass spectrometry; ML: machine learning; MS: mass spectrometry; *m/z*: mass to charge ratio; NCE: normalized collision energy; PTM: posttranslational modification; PX: ProteomeXchange.
