# Supplementary material for: Variability analysis of LC-MS experimental factors and their impact on machine learning
Source: Gigascience. 2023 Nov 20;12:giad096. doi: 10.1093/gigascience/giad096 (PMC10659119; doi:10.1093/gigascience/giad096)
Supplement: giad096_Supplemental_File [file giad096_supplemental_file.pdf]

Supporting Information for Publication.

Title: Variance Analysis of LC-MS Experimental Factors and Their Impact on Machine Learning.

Authors: Tobias Greisager Rehfeldt[1], Konrad Krawczyk[1], Simon Gregersen Echers[2], Paolo Marcatili[3], Richard Röttger[1], Veit Schwämmle[4]

Table of Content

Supplementary Figure 1. **2**

Supplementary Figure 2. **2**

Supplementary Figure 3. **3**

Supplementary Figure 4. **4**

Supplementary Figure 5. **4**

Supplementary Figure 6. **5**

Supplementary Figure 7. **6**

Supplementary Figure 8. **7**

Supplementary Table 1. **8**

Supplementary Table 2. **9**

Supplementary Table 3. **9**

Supplementary Table 4. **9**

Supplementary Table 5. **9**

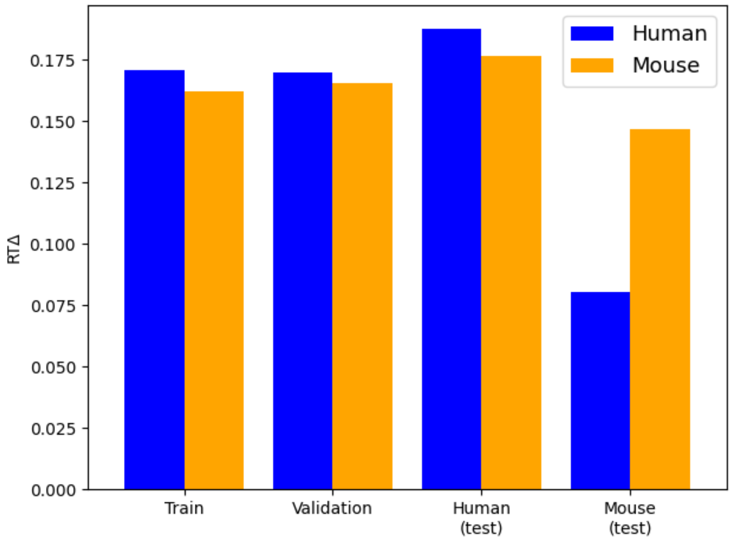

Supplementary Figure 1.

Species model performance comparison. Each model was trained and validated on individual internal datasets and cross-tested on all external datasets as a basis for model comparisons. The x-axis denotes datasets, and the bars denote model performance on the given dataset.

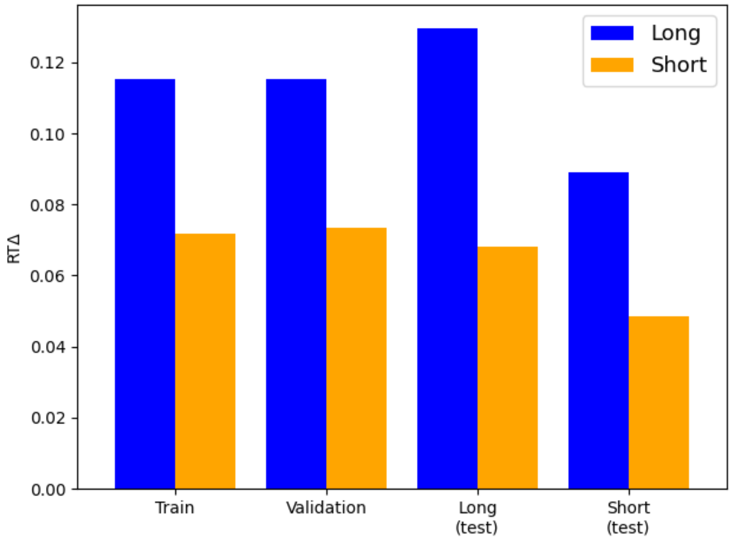

Supplementary Figure 2.
Gradient model performance comparison. Each model was trained and validated on individual internal datasets and cross-tested on all external datasets as a basis for model comparisons. The x-axis denotes datasets, and the bars denote model performance on the given dataset.

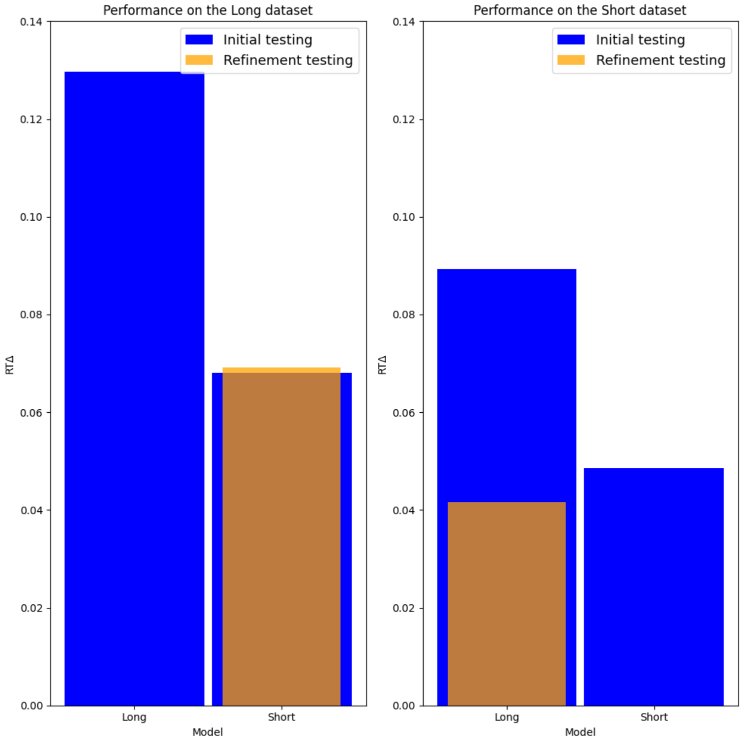

Supplementary Figure 3.
Gradient-based refinement model performance comparison. Each model was refined on all previous external datasets and re-tested on both datasets. Datasets are separated by plots, denoting the performance difference of each model when trained on, or refined to, identical datasets. Each bar has the original test metric in blue, and the refinement test metric overlaid in orange.

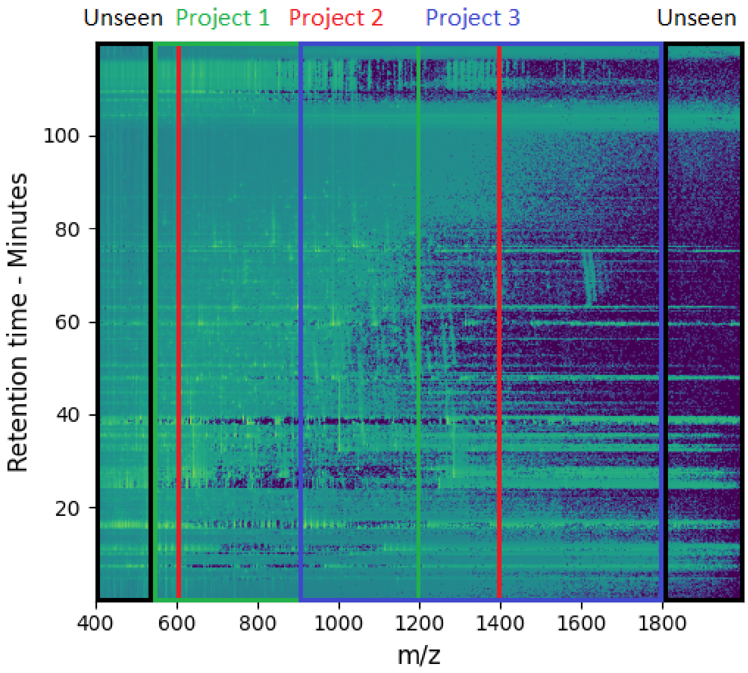

Supplementary Figure 4.
Illustration of the unseen data space caused by limited search space filters. Illustrated are three theoretical search spaces superimposed on top of a theoretical MS1 chromatogram, with unreported peptides at both ends of the data space.

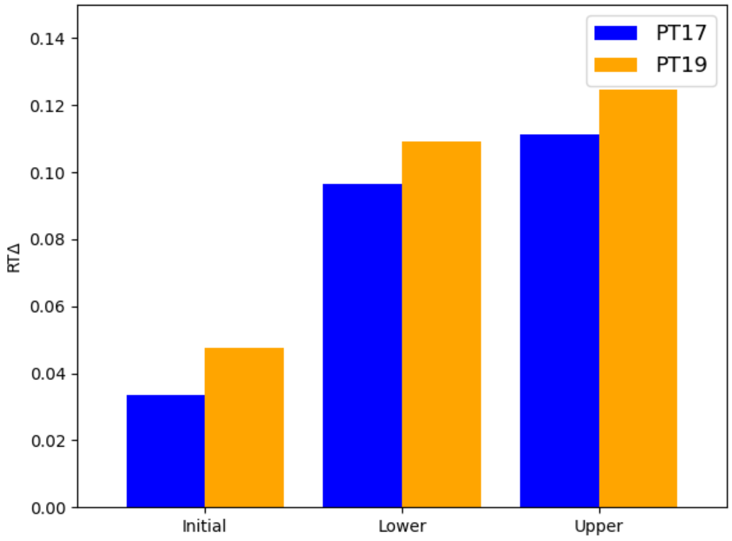

Supplementary Figure 5.
Out-of-bounds performance comparison of PT models. The *PT17* and *PT19* model comparison for their individual internal datasets (Initial), peptides with m/z below 360 (Lower) and above 1300 (Upper).

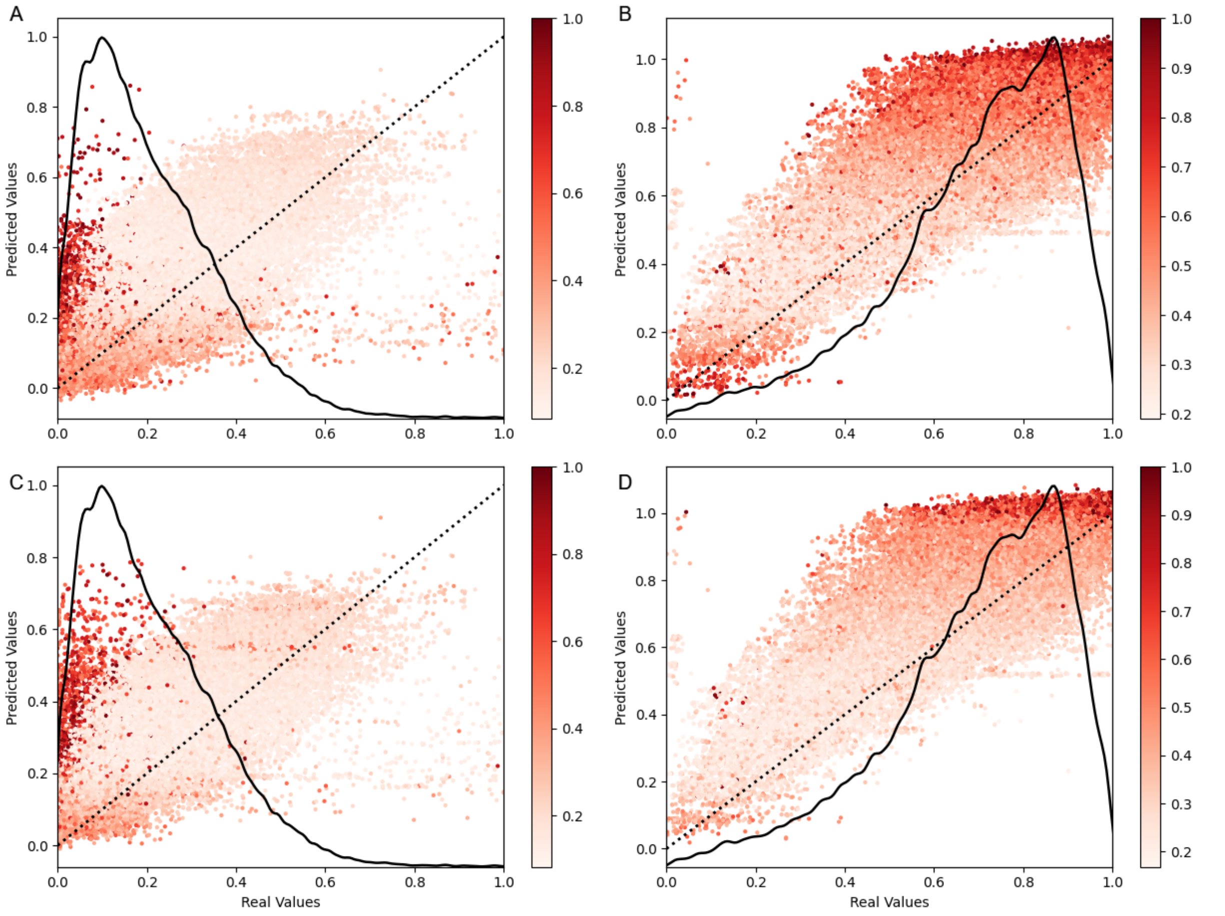

Supplementary Figure 6.

Bayesian model uncertainty estimates for out-of-bounds peptides split into two datasets. The out-of-bounds datasets are split into two datasets: peptides with m/z below 360 (Left) and above 1300 (Right). Models are separated into rows with *PT17* in the first row (A, B) and *PT19* in the last row (C, D).

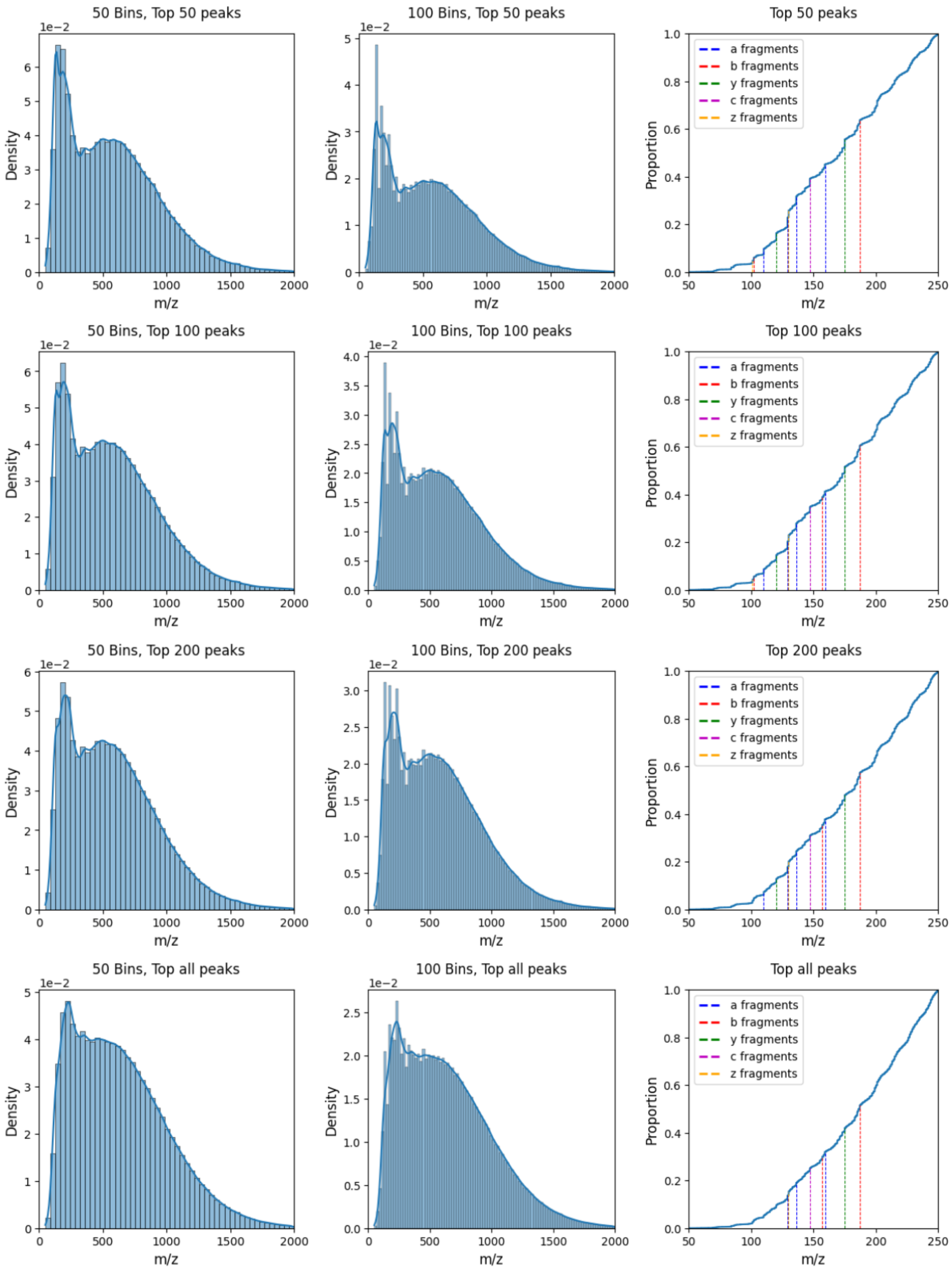

Supplementary Figure 7.

Density distribution of MS2 peaks with different levels of peak picking imposed at 50 and 100 bins. For each value of peak picking, we also illustrated the cumulative distribution plot of the peaks in 50-250 m/z with single AA fragmentations overlaid

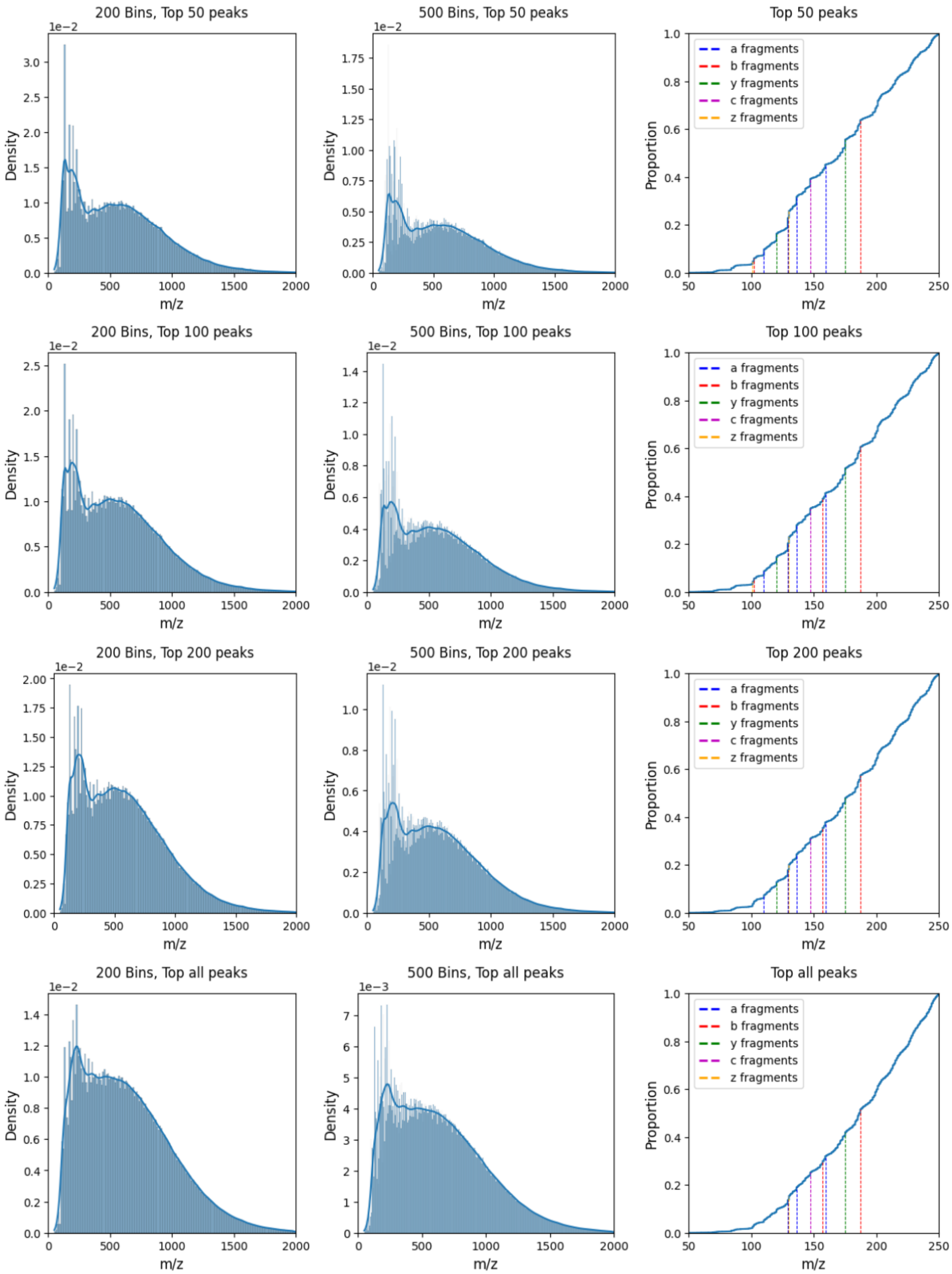

Supplementary Figure 8.

Density distribution of MS2 peaks with different levels of peak picking imposed at 200 and 500 bins. For each value of peak picking, we also illustrated the cumulative distribution plot of the peaks in 50-250 m/z with single AA fragmentations overlaid

m/z	Amino Acid(s) and Fragmentation	Frequencies *(50, 100, 200, all)*
101.1	Glutamine *(a)* & Lysine *(a)*	1.15%, 1.17%, *, *
102.1	Glutamate *(a)* & Threonine *(b)*	1.23%, 1.14%, *, *
110.1	Histidine *(a)*	2.55%, 1.83%, 1.33%, *
120.1	Phenylalanine *(a)* & Threonine *(y)*	3.14%, 2.17%, 1.56%, *
129.1/129.2	Glutamine *(a)* & Lysine *(b)* & Arginine *(a)*	4.13%, 3.08%, 2.39%, 1.57%
130.1	Glutamate *(b)* & Glutamine *(z)* & Lysine *(z)*	2.89%, 2.62%, 2.3%, 1.69%
136.1	Tyrosine *(a)*	3.37%, 2.45%, 1.81%, 1.18%
147.1	Glutamine *(y)* & Lysine *(y)* & Glutamate *(c)*	3.19%, 2.31%, 1.74%, 1.21%
157.2	Arginine *(b)*	*, 1.18%, 1.31%, 1.16%
159.2	Tryptophan *(a)*	1.46%,'1.49%, 1.47%, 1.19%
175.2	Arginine *(y)*	3.54%, '2.7%, 2.09%, '1.47%
187.2	Tryptophan *(b)*	1.62%, 1.75%, 1.8%, 1.57%

Supplementary Table 1.

Corresponding single amino acid peak table. m/z, amino acid and fragmentation type of MS2 peaks on Figure 9. ‘*’ does not meet the threshold of 1% in the given *n,* and is not annotated on the CDF. *Note that 129.1 and 129.2 have been merged to one peak, due to rounding error.*

		Models				
		PT17	*PT19*	*Limit*	*Wide*	**Means**
**Datasets**	*PT17*	100.0%	60.7%	119.7%	154.1%	108.6%
	PT19	64.2%	100.0%	188.7%	150.9%	126.0%
	Limit	60.7%	77.0%	100.0%	41.0%	69.7%
	Wide	51.2%	68.6%	74.4%	100.0%	73.6%
						94.5%

Supplementary Table 2.
Model epochs for training convergence overview of models trained in section r.1.

		Models		
		Long	*Short*	**Means**
**Datasets**	*Long*	100.0%	106.9%	103.5%
	Short	192.9%	100.0%	146.5%
				125.0%

Supplementary Table 3.
Model epochs for training convergence overview of models trained in section r.2.

		Models		
		Human	*Mouse*	**Means**
**Datasets**	*Human*	100.0%	103.1%	101.6%
	Mouse	45.3%	100.0%	72.7%
				87.1%

Supplementary Table 4.
Model epochs for training convergence overview of models trained for organisms.

		Models		
		Orbitrap	*Q. Exactive*	**Means**
**Datasets**	*Orbitrap*	100.0%	156.4%	128.2%
	Q. Exactive	157.1%	100.0%	128.6%
				128.4%

Supplementary Table 5.
Model epochs for training convergence overview of models trained for instruments.
