# Supplementary material for: Variability analysis of LC-MS experimental factors and their impact on machine learning
Source: Gigascience. 2023 Nov 20;12:giad096. doi: 10.1093/gigascience/giad096 (PMC10659119; doi:10.1093/gigascience/giad096)
Supplement: giad096_GIGA-D-23-00094_Revision_1 [file giad096_giga-d-23-00094_revision_1.pdf]

GigaScience

Variability Analysis of LC-MS Experimental Factors and Their Impact on Machine Learning

--Manuscript Draft--

**Manuscript Number:**	GIGA-D-23-00094R1
**Full Title:**	Variability Analysis of LC-MS Experimental Factors and Their Impact on Machine Learning
**Article Type:**	Research
**Funding Information:**	Velux Fonden (00028116) — Mr Veit Schwämmle
**Abstract:**	Abstract<br>Background<br>Machine learning (ML) technologies, especially deep learning (DL), has gained increasing attention in predictive mass spectrometry (MS) for enhancing the data processing pipeline from raw data analysis to end-user predictions and re-scoring. ML models need large-scale datasets for training and re-purposing, which can be obtained from a range of public data repositories. However, applying ML to public MS datasets on larger scales is challenging, as they vary widely in terms of data acquisition methods, biological systems, and experimental designs.<br>Results<br>We aim to facilitate ML efforts in MS data by conducting a systematic analysis of the potential sources of variability in public MS repositories. We also examine how these factors affect ML performance and perform a comprehensive transfer learning to evaluate the benefits of current best practice methods in the field for transfer learning.<br>Conclusions<br>Our findings show significantly higher levels of homogeneity within a project than between projects, which indicates that it's important to construct datasets most closely resembling future test cases, as transferability is severely limited for unseen datasets. We also found that transfer learning, although it did increase model performance, did not increase model performance compared to a non-pre-trained model.
**Corresponding Author:**	Veit Schwämmle<br><br>DENMARK
**Corresponding Author Secondary Information:**	
**Corresponding Author's Institution:**	
**Corresponding Author's Secondary Institution:**	
**First Author:**	Tobias Greisager Rehfeldt
**First Author Secondary Information:**	
**Order of Authors:**	Tobias Greisager Rehfeldt<br>Konrad Krawczyk<br>Simon Gregersen Echers<br>Paolo Marcatili<br>Pawel Palczynski<br>Richard Röttger<br>Veit Schwämmle
**Order of Authors Secondary Information:**	
**Response to Reviewers:**	-----------<br>We want to thank the reviewers for their excellent feedback, allowing us to improve the

Powered by Editorial Manager® and ProduXion Manager® from Aries Systems Corporation

quality of the reports and writing in the paper.

Reviewer reports:
Reviewer #1: This paper aimed to facilitate machine learning efforts in mass spectrometry data by conducting a systematic analysis of the potential sources of variance in public mass spectrometry repositories. This paper examined how these factors affect machine learning performance and performed a comprehensive transfer learning to <evaluate the benefits of current best practice methods in the field for transfer learning. Although the experimental content is extensive and provides promising results, some <major points need to be addressed as follows:

Please explain the rationality of the RT used for evaluating model performance. In addition, it is necessary to increase other evaluation metrics to provide a more powerful comparison of model performance.

ΔRT is the metric used by the creators of the DLOmix package from the Lab which created the Prosit model, which is why we decided to use this metric. This explanation has also been added to the manuscript. However, during training, we also monitored MSE and MAE which we have added to the paper for further comparison. While MSE often showed more significant differences between training and testing, all models exhibited the same patterns regardless of the metric used for evaluation. We have added an explanation for the choice of a metric within the manuscript, as well as added an "Evaluation Metric Report.pdf" file which contains every model performance for both ΔRT, MSE, and MAE, illustrating performance invariance across metrics.

The curves in Figures 6 and 8 should provide more explanations to help readers understand. In addition, all figures are somewhat blurry and clearer figures should be provided.

We have updated the figure text for both figures 6 and 8 to help readers understand. The images we provided to GigaScience were not of low quality or DPI. We suspect that the quality loss was produced during the conversion of the uploaded files.

This paper does not provide specific implementation steps of variance. Please describe the variance analysis process in mathematical language and provide the corresponding mathematical formula.

We apologize for using a slightly misleading expression. We never intended to use "variance" in the mathematical sense but more as a surrogate or heuristic of variability found within the data. As complex LC-MS data has no meaningful way of computing mean or variance values we are incapable of providing single values for variance in any meaningful sense. Instead, we have replaced the word "variance" with "variability" throughout the manuscript.

There are some formatting issues: Keywords and the title 'Data Description' should only have the first letter capitalized. On pages 6, 17, and 18, the font size of the article is inconsistent.

Thank you for pointing this out. We fixed the capitalization of the Data description and certain keywords. The inconsistent font and font size were not present in the document we sent to GigaScience. Why the text was inconsistent when the reviewers received them, we do not know.

There are some grammar issues: On pages 6 and 16, dataset should be added with 's'. On page 7, lines 9-10, the tense is not unified.

We fixed a few cases of datasets either having or missing and ‘s’ at the end. We also fixed the tense issue on page 7

There are significant issues with the format of references. Inconsistent capitalization of initial letters in literature titles, such as [1] and [5]; Some literature lacks page numbers, such as [6] and [18]. Please re-organize the references according to the format required by the journal.

We have recognized errors in the references section and fixed some errors with capitalization and page numbers. However, according to the GigaScience submission rules, we should not apply Title Case to references and have altered all titles to be as they were initially published.

Reviewer #2: The paper applies machine learning to publicly available proteomics data sets and assesses the ability to transfer learning algorithms between projects. The primary aim of these algorithms appears to be an attempt to increase consistency of retention time prediction for data-dependent acquisition data sets, however this is not explicitly stated within the text. The application of machine learning to derive insight from previous performed proteomics experienced is a worthwhile exercise.

The authors report ΔRT to determine fitting for their models. It would be interesting to see whether the models had other metrics used to assess fitting, or could be used to increase number of identifications within sample sets, and whether this was successful. ALternatively, was there any conclusions able to be drawn about peptide structure and RT determination from these models?

We thank the reviewer for this important point. It was also brought up by reviewer 1, so we will append the same response here: ΔRT is the metric used by the creators of the DLOmix package from the Lab which created the Prosit model, which is why we decided to use this metric. However, during training, we also monitored MSE and MAE which we have added to the paper for further comparison. While MSE often showed more significant differences between training and testing, all models exhibited the same patterns regardless of the metric used for evaluation. We have added an explanation for the choice of a metric within the manuscript, as well as added an “Evaluation Metric Report.pdf” file which contains every model performance for both ΔRT, MSE, and MAE, illustrating performance invariance across metrics.
Due to constraints during the project and the extensive training of the models, we were, unfortunately, unable to do tests on peptide structure.

Project specific libraries are well known to improve results compared with publicly available databases, and the discussion on this point should be developed further through comparison of this work with other papers - particularly with advances in machine learning and neural networks in the data independent analysis field.

This is an important point. We have added a short section in the “fragmentation pattern” section where we discuss project specific library’s ability to improve peptide identifications while also highlighting how such libraries can bias the datasets. However, as our work does not consider the efficacy of peptide identifications, but rather the variability of the peptide elutions, we do not believe that it would merit a larger change of the manuscript.

Comparison of Q-Exactiv models vs Orbitraps appears to be somewhat redundant, and possible a result of poor meta-data as Q-Exactiv instruments are orbitrap mass spectrometers. A more interesting comparison to make here would be between orbitrap and TOF instruments, though as the datasets have all been processed through

MaxQuant, it is likely the vast majority were acquired on orbitrap instruments.

We agree with the reviewer on this point and have opted to remove the Q. Exactive vs Orbitrap model comparison from the manuscript as well as the supplementary material. However, during data extraction, we were unable to acquire an adequate amount of ToF data points to conduct any meaningful statistics. This results in almost all data being either orbitrap or Q. Exactive instruments, at a relatively equal split.

The paper uses ΔRT as the readout for all models tested, however the only chromatography variable considered in testing the models is gradient length. However, chromatography is also dependent on column chemistry, column dimensions, composition of buffer, use of traps, temperature etc. These are also likely to be contributing the variance observed between the PT datasets where these variables will be consistent and publicly available datasets. These factors are also likely to play a role in higher uncertainty for early and late eluting peptides where these factors are likely to vary most between sample sets. The metadata may not be available to use to compare within the data sets selected, so the authors should at minimum make discussion around these points.

We agree with the reviewer, that we lack a proper discussion of other variability-inducing factors in the LC-MS system setup. These factors are difficult to obtain and we thus
omitted investigating their impact. We have added new paragraphs which discuss these factors in detail, and why we are unable to train models or compute statistics with these factors.

Sample preparation is likely to have similar effects, and as the PT datasets are generated synthetically using ideal peptides, publicly available datasets will be generated from complex sample mixtures, and have increased variance due to inefficiencies of digestion, sample clean up and matrix effects. Previous studies on variance have also described sample preparation as the highest cause of variance. This needs further discussion

----
This point extends on the previous point, and we have extended the section on LC-MS system variability concerns to also encompass sample preparation. This section highlights the problem of unaccountable factors which are often under-reported and can not extracted from the raw files.

While the isolation windows of the m/z will lead to unobserved space, search engines setting will also apply here. From the text, it appears that the only spectra that were considered were those already identified in a search program (due to having Andromeda cut-off scores always apply). Typical setting for a database search will have a cut off of peptide sequences of at least 7 residues, making peptide masses appearing lower than 350 m/z unlikely. There is also significant amount of noise below 350 m/z and this also likely contributes to poorer fitting.

We agree with the reviewers' points here and have added a short paragraph to section 3 which mentions database search cut-offs and noise in the lower end of the m/z space.

The authors identify differences in MSMS spectral features, however, most of these points are well known in the field. The authors should develop the discussion on the causes of the differences in fragmentation, as CID low mass drop off is expected, and the change in profile is expected with increasing activation energies. A more developed analysis could exclude precursor masses from these plots and focus solely on fragment ions generated.

	-----------<br>We agree that the fragmentation technique and energy section comprised too many confusing and misleading methodologies, and did not align well with the retention time predictor-based methodology we have used throughout the manuscript. For this reason, we have entirely removed this part of the fragmentation section, along with the 6 figures in the supplementary material. This section has instead been replaced by a paragraph that describes how fragmentation technique/energy impacts the MS2 spectra, and how this information is used in ML.<br>-----------<br><br>The authors highlight that internal fragmentation of peptides could be used as a valuable resource to implement in machine learning. There has already been some success using these fragmentation patterns for sequence identification within both top-down and bottom up proteomic searches that the authors should consider discussing. However, <these data do not appear to be incorporated into the machine learning models in this paper - or at least seem not to play a significant role in prediction, and this section <appears to be a bit out of place.<br><br>-----------<br>As we removed the majority of this section, most of this comment is no longer relevant. We did, however, discuss and add references for the usage of fragmentation patterns in ML in proteomics.<br>-----------
**Additional Information:**	
**Question**	**Response**
Are you submitting this manuscript to a special series or article collection?	No
**Experimental design and statistics**<br><br>Full details of the experimental design and statistical methods used should be given in the Methods section, as detailed in our Minimum Standards Reporting Checklist. Information essential to interpreting the data presented should be made available in the figure legends.<br><br>Have you included all the information requested in your manuscript?	Yes
**Resources**<br><br>A description of all resources used, including antibodies, cell lines, animals and software tools, with enough information to allow them to be uniquely identified, should be included in the Methods section. Authors are strongly encouraged to cite Research Resource Identifiers (RRIDs) for antibodies, model organisms and tools, where possible.	Yes

Powered by Editorial Manager® and ProduXion Manager® from Aries Systems Corporation

Have you included the information requested as detailed in our Minimum Standards Reporting Checklist?	
**Availability of data and materials**<br><br>All datasets and code on which the conclusions of the paper rely must be either included in your submission or deposited in publicly available repositories (where available and ethically appropriate), referencing such data using a unique identifier in the references and in the “Availability of Data and Materials” section of your manuscript.<br><br>Have you have met the above requirement as detailed in our Minimum Standards Reporting Checklist?	Yes

Powered by Editorial Manager® and ProduXion Manager® from Aries Systems Corporation

Manuscript Click here to access/download;Manuscript;Paper Changed.docx

Variability Analysis of LC-MS Experimental Factors and Their Impact on Machine Learning

Tobias Greisager Rehfeldt, Konrad Krawczyk, Simon Gregersen Echers, Paolo Marcatili, Pawel Palczynski, Richard Röttger, Veit Schwämmle

Abstract

Background

Machine learning (ML) technologies, especially deep learning (DL), has gained increasing attention in predictive mass spectrometry (MS) for enhancing the data processing pipeline from raw data analysis to end-user predictions and re-scoring. ML models need large-scale datasets for training and re-purposing, which can be obtained from a range of public data repositories. However, applying ML to public MS datasets on larger scales is challenging, as they vary widely in terms of data acquisition methods, biological systems, and experimental designs.

Results

We aim to facilitate ML efforts in MS data by conducting a systematic analysis of the potential sources of variability in public MS repositories. We also examine how these factors affect ML performance and perform a comprehensive transfer learning to evaluate the benefits of current best practice methods in the field for transfer learning.

Conclusions

Our findings show significantly higher levels of homogeneity within a project than between projects, which indicates that it's important to construct datasets most closely resembling future test cases, as transferability is severely limited for unseen datasets. We also found that transfer learning, although it did increase model performance, did not increase model performance compared to a non-pre-trained model.

Keywords

Machine learning, Deep learning, Data mining, Statistics, Bioinformatics, Proteomics, Mass spectrometry, Transfer learning

Background

Large-scale studies of proteomes are essential to our understanding of the biological processes within an organism. The leading technology for characterizing thousands of proteins is Liquid-Chromatography Mass Spectrometry (LC-MS), which enables high-throughput quantification of protein abundances in a biological sample [1,2] (Figure 1).

Figure 1. Simplified workflow of a Mass Spectrometry-based proteomics experiment. First, proteins are extracted from the biological samples, after which they are digested into peptides using enzymes, most often trypsin. Next, peptides are chromatographically separated and injected into the mass spectrometer where they are measured according to the mass over charge (m/z) and abundance (MS1). MS1 spectra from all peptide precursor ions are reported, and certain peptides are chosen for tandem-mass spectrometry (MS2), where they are fragmented along their amino acid backbone and identified by having their MS2 spectrum matched to a database of theoretical spectra. Lastly, peptides are quantified and summarized into proteins.[3–5]. Created with BioRender.com.

LC-MS has become the standard within proteomics procedures and continues to generate vast amounts of data which, due to increasing demands from journals and reviewers, is often made publicly available in data repositories. This change has led to numerous public data sets being registered in online repositories such as the ProteomeXchange (PX) consortium [6]. The PXC contains references to over 17.000 projects, and its largest member, PRIDE, has more than a million raw files. Each raw file contains an average of 6.778 MS1 and 32.016 MS2 spectra, which amounts to over 39 billion mass spectra. These data repositories provide an invaluable resource for data repurposing to address novel biological questions or to benchmark new computational techniques for proteomics data analysis.

While efforts in harmonizing data accessibility within ProteomeXchange and standardizing the computational pipelines are ongoing [6], repurposing data from these repositories comes with a significant entry barrier, as they do not yet have any systematic criteria for metadata or data types.

Due to the advancements in machine learning (ML) model development, there is now an increasing interest in repurposing this rich LC-MS data to train complex ML models that can produce new insights and results not achievable by previous computational methods [7]. However, the large diversity of experimental procedures and biological systems requires careful consideration when applying bioinformatics methods to larger extracts of publicly available data as ML relies on careful balancing to reach optimal and correct performance.

Multiple ML algorithms and methods have been applied to MS data, such as regression models [8], random forest [9], and more recently neural networks [10]. Machine learning applications in proteomics are primarily focused on two aspects; (1) improving current methodologies such as database searches or de-novo sequences, or (2) predicting physico-chemical peptide properties such as LC-MS/MS spectra, retention time, or post-translational modifications (PTMs) [11–13]. Deep learning (DL) approaches function by generalizing the data, thereby generating distributions of the training data. However, due to the high complexity and large noise found in LC-MS data, many of the current approaches suffer from limited transferability, as they utilize synthetic, limited, or heavily stratified datasets for the purpose of training and testing their models [11,14,15]. These issues are further exacerbated by technical advances in the field such as ion mobility [16], which further increases the complexity and diversity of the data. The majority of current ML methods within computational proteomics also rely on unique and complex post-processing pipelines, such as peptide-specific indexed retention times (iRT) calculations, rendering the methods difficult to replicate and reducing their application range outside the original publication.

One of the biggest shortcomings in machine learning, and particularly deep learning, is the problem of under and overfitting. These refer to situations in which a model either performs too well on the training data (overfitting) and generalizes poorly on unseen test data, or not well enough on the training data (underfitting) and subsequently also on unseen test data. Despite multiple attempts and the breadth of available data, these problems are still present in the field of predictive proteomics.

In this manuscript, we investigate the general reusability of public mass spectrometry data for machine learning applications, with a specific focus on potential pitfalls that could result in poor translatability to independently sampled data sets. We will do so by performing statistical analyses on the effect of the experimental setups on the variability of the generated data, and see how these effects impact the predictive capabilities of state-of-the-art deep learning models. This work is expected to have an impact on the data selection process in predictive proteomics, elevating the capabilities of current and future models, as well as highlighting the necessity for appropriate pre-processing and algorithmic choices.

Data description

For a comprehensive representation of publicly available MS data, we analyzed data from ~60.500 raw files across ~820 PRIDE projects, totaling ~60 TB of raw files and metadata. 546 projects containing 33.426 raw files were used for neural network testing. All selected data had been previously analyzed with MaxQuant [17].

Figure 2. Andromeda score (MaxQuant) distribution plot of the ~151M unmodified peptides in the complete data.

The full dataset was gathered from randomly sampled projects on PRIDE using MS2AI [18]. We restricted the retrieval to data from standard bottom-up proteomics experiments. We also did not have any initial queries on experimental or sample preparation, resulting in data from a wide breadth of sources from which we have sub-sampled smaller datasets for in-depth analyses (Table 1). In total, we gathered spectra and metadata for ~151M individual unmodified peptides (Figure 2).

Table 1. Overview of datasets used in model training or testing. All subset datasets are randomly sampled from the full ~151M peptide dataset, with Andromeda score and subset filters described in the "Minimal score" and "Description" columns respectively, no other filters were added when sampling. We also annotated the size of the subset dataset in the "Peptide count" column, with a 2M peptide count limit for each dataset.

Name	Section	Minimal score	Peptide counts	Description
PT17	1	100	750.000	Dataset constructed from PXD004732
PT19	1	100	750.000	Dataset constructed from PXD010595
Limit	1	100	750.000	750.000 peptides excluding PXD004732 and PXD010595
Wide	1	150	2.000.000	2.000.000 peptides excluding PXD004732 and PXD010595
Long	2	150	2.000.000	Gradient length equal to or above 100 minutes
Short	2	150	2.000.000	Gradient length equal to or below 60 minutes
Lower	3	100	125.000	Peptides with m/z values below 360
Upper	3	100	125.000	Peptides with m/z values above 1300
Human	Supp.	150	2.000.000	“Human (Homo Sapiens)” from PRIDE metadata
Mouse	Supp.	150	2.000.000	“Mus musculus (mouse)” in PRIDE metadata

Results and discussions

We performed a thorough statistical assessment of the data and trained multiple neural networks, in order to gauge the variability and evaluate the transferable capabilities of state-of-the-art models. However, in the field of predictive proteomics, and especially in retention time prediction, it is common to apply transfer learning to pre-trained models. This is done as a result of the poor transferability of the original networks, as the models do not achieve setup independence by being constrained to the experimental settings of the training data. However, transfer learning requires a large amount of data in a format suitable for machine learning, as well as significant computational expense, making it both data- and computationally-intensive. Additionally, it also requires expertise in both machine learning and programming. In order to test the effectiveness of transfer learning in predictive proteomics, we exhaustively transferred all of the trained models to every other dataset in the same section to assess the impact of transfer learning on performance.

All models were measured with several metrics, namely RTΔ, mean squared error (MSE), and mean absolute error (MAE) (Supplementary evaluation_metric_report.pdf). However, as the model performances indicate metric invariance, and therefore do not change the conclusions drawn from the model performance, we only report the RTΔ values. RTΔ measures the average time difference between predicted RT values and actual values, and is the proprietary metric used by the DLOmix package. DLOmix is a software integration of popular DL models used in proteomics, which we used to construct the Prosit model.

In this manuscript, we use the transferability of model predictions as a measure of data variability by training models to predict the elution times of identified peptides based on a range of measurable factors. And while we try to stratify datasets to keep certain parameters constant, there is a range of under-reported and unextractable information that we are incapable of controlling which are often introduced from the sample preparation or the LC system.

During sample preparation, the efficiency of protein digestion can cause incomplete digestion or non-specific cleavage which can result in the presence of partially digested peptides. These altered peptide species can significantly influence the elution behavior, adding complexity and variability to the analysis. Additionally, sample cleanup protocols are employed to remove interfering impurities like salts and detergents, which, based on the efficiency of the procedure, may leave behind residual impurities that can affect the peptide elution process. Moreover, the sample matrix composition introduces the matrix effect, which can impact peptide ionization and detection.

In the LC system, column chemistry determines the interactions between peptides and the stationary phase, while column dimensions affect separation efficiency and resolution. Modifying either the mobile or stationary phase composition can alter the peptide's polarity, ionic strength, binding affinity, etc., altering the elution time and profile. The use of traps or pre-columns in the LC system introduces variability by selectively retaining peptides or interfering with separation. LC Temperature influences molecular interactions, including solvent viscosity, peptide conformation, and peptide-solvent interactions, thereby affecting peptide elution and separation.

These are just some of the unextractable factors that can contribute to the variability and affect the elution times of peptides. Unfortunately, we are unable to measure or control these factors. However, by having a sufficiently large dataset and randomly drawing data points from a range of thousands of raw files, we aim to mitigate their impact on the model predictions and conclusions, also by assuming that the majority is based on standard LC setups.

1 Single vs multi-project variability

In our model comparisons, we found that the models trained on the *PT17* and *PT19* datasets performed significantly better than those models trained on the *Limit* and *Wide* datasets during both training and validation (Figure 3). Interestingly, the *PT* models also outperformed the *Wide* model, and performed comparably to the *Limit* model, when testing on the wide and limited datasets.

While we expected the training and validation of the *PT* models to outperform the *Wide* and *Limit* model, we did not expect the *PT* models to outperform the *Wide* and *Limit* models on the *Wide* and *Limit* test datasets. Furthermore, we also found that the *Limit* model outperformed the *Wide* model for all test cases, suggesting that increasing the amount of data and using stricter scoring criteria does not necessarily improve the performance of trained models, and may even cause the models to underfit. One possible explanation for this is that longer and more complex peptides, which are easier to detect and in general receive higher peptide identification scores, also exhibit more variability in their elution times. We tested this and found that the peptides in both *PT* datasets have average peptide lengths of ~12, with the *Limit* dataset containing a longer average peptide length of ~13, and the *Wide* dataset containing an even longer average length of ~14.

It is also possible that the reduced performance observed in the randomly sampled datasets is due to the presence of multiple variability-inducing factors in the experimental setup. These factors, such as the type of MS instrument or the selected species, are often difficult or impossible to account for when relying on large bulks of public data. Changing these could result in considerably different model performances (Supplementary Figure 1). In contrast, the *PT* datasets were measured under the same conditions on

synthetic peptides, which reduces the presence of such variability-inducing factors. Additionally, the *PT* datasets have the advantage of using one single gradient length, while the limited and wide datasets use multiple different gradient lengths.

Figure 3. General variability model performance comparison. Each model was trained and validated on its original source datasets and then cross-tested for all test datasets. We compared the training and validation for all models, as well as cross-testing datasets and their respective model performance in terms of RTΔ

In order to evaluate their transferability, we applied transfer learning to all four models and found that, while some of the models improved performance compared to previous external testing, they mostly performed similarly to non-transferred models on the same dataset (Figure 4). In the case of the *Wide* dataset, transfer learning actually resulted in reduced performance for all transferred models. This suggests that the *Wide* dataset had significantly higher levels of heterogeneity between training and testing data compared to the other datasets. While these results show that transfer learning can be beneficial in certain scenarios, they also showed that most of the models simply improved or regressed to the transfer dataset. However, even if transfer learning did not provide significant predictive benefits, it did reduce the time needed to train the models by an average of 5.5% by converging faster (Supplementary Table 2).

Figure 4. General variability transfer model performance comparison. Each model was transferred to each of the external datasets and re-tested on all four datasets. Datasets are separated by plots, denoting the performance difference of each model when trained on or transferred to identical datasets. Each bar has the original test metric in blue, and the transfer learning test metric overlaid in orange.

2 Gradient lengths

The retention time of a peptide in an LC-MS/MS system is determined by the interactions between the peptide and the stationary and mobile phases of the liquid chromatography system. In identical setups, the retention time of a peptide is considered reproducible[8].

Plotting the distribution of gradient length for all raw files with an overlaid cumulative distribution function (Figure 5a), we observe significant peaks at 60, 90, and 120 minutes, with ~60% of all gradients being 0–120 minutes in length and the longest gradient being 800 minutes. While we did find single projects with as many as 15 different gradients, we also found that 70% of the 820 projects kept the same gradient length for all files, while only ~5% employed more than two unique gradients (Figure 5b), indicating high levels of consistency in instrument configurations within individual projects.

Figure 5. Gradient length distribution and unique gradient per project. We illustrated the gradient distribution of all 60.000+ raw files overlaid with the cumulative distribution (A). Also illustrated is the number of unique gradients found within any of the 820 projects (B)

The results of our deep learning use case showed that the *Short* gradient model performed significantly better than the *Long* gradient model (Supplementary Figure 2). This is further supported by its decreased performance on the *Long* gradient test dataset compared to the *Short* model.

These findings suggest that peptides from longer gradients generally express higher variability compared to peptides from shorter gradients, even after attempted peptide normalization. It also indicates that our normalization method for the effective gradient, which aims to mimic the linear iRT calculations used in the original Prosit paper, may not be effective for all gradients and raw files, reiterating the necessity of targeted post-processing pipelines.

Performing inference dropout on all models in the previous section shows that all of the models exhibit significantly higher uncertainty for the earliest and latest eluted peptides compared to those eluted closer to the middle of the gradient (Figure 6). Additionally, we observe that the *PT* models show a more linear prediction gradient than the *Limit* and *Wide* models, further suggesting the controlled nature of the ProteomeTools datasets outputs peptides in a more linear gradient, which fits better for our first-last peptide gradient normalization. We also observe less overall uncertainty in the PT models, likely due to the fact that they were trained on datasets with fewer peptides, lower average peptide retention times of approximately 32 minutes, and unified gradients, whereas the average retention times of the limit and

wide datasets were significantly higher at 60+ minutes from multiple gradient lengths. This suggests that longer gradients lead to an increase in data variability and model uncertainty.

Figure 6. Bayesian model uncertainty estimates of single vs multi-project variability models. For each of the models in section 1, we conducted model inference 25 times with layer dropouts applied at original rates. Each data point is an average of the inference results, with the colorization of the dots indicating the normalized relative variability of each peptide. For each of the datasets, we overlaid a distribution plot of the dataset retention time values as a solid line, and a linearity assessment (y=x) to facilitate a comparison of the model predictions with perfect alignment as a dotted line.

Performing transfer learning on the gradient length models (Supplementary Figure 3) we observe that refining the *Long* model to the *Short* dataset resulted in a significant performance improvement. However, refining the Short model to the Long dataset did not result in any significant change in performance, although it still outperformed its non-transferred counterpart. Unlike what we observed in the previous section, transfer learning of the gradient length models came at an increase or stagnation in performance, indicating that the models retained information from the initial training datasets. We also note that transferred models took on average 25% longer to train compared to non-transferred models (Supplementary Table 3).

3 Mass-to-charge range filter

MS instruments have a range of setup parameters that can tailor the experiment to the needs of researchers. The m/z range filter is one of those parameters, as it restricts peptide data acquisition to a given m/z range. However, for data re-purposing, this range can also lead to a biased dataset for machine learning, as the sample might have contained a large range of peptides not reported by the instrument. While the filter settings do exclude certain data points, it should be noted that most database searches also have cut-offs for shorter peptides due to noise at the lower end of m/z, making peptide identifications in this space more unlikely even when peptides are present.

When plotting the m/z filters of the mass acquisition range (Figure 7), we observe significantly more variability in the upper bound compared to the lower bound, meaning that our upper bound is highly

correlated to the length of the filter. All violin plots exhibit a peak at one specific value, 350 for the lower bound, and 1500 for the upper bound, with a corresponding peak at 1150 for the filter lengths. These peaks correspond to the most commonly used filter which accounts for 33.9% of all raw files.

Figure 7. **Illustration of the variability of m/z range filters found in different projects**. Violin plots depicting the m/z filter bound distributions, with the lower bound plotted in blue (A), upper bounds plotted in red (B), and filter length plotted in yellow (C). A histogram of the total number of unique filters found within projects (D).

We also observed that 47% of all projects applied a single filter across all raw files, 43% of projects applied two filters and only 10% of the projects applied more than two unique filters. Consequently, there is significant homogeneity within a project while between projects the filters can differ considerably. If datasets are constructed using only one or a few unique filters, large portions of the data space may never be used for training, potentially limiting the transferability of the models (Supplementary Figure 4).

We also tested the model performance of the *PT17* and *PT19* models on peptide datasets only containing peptides outside of the original filter bounds (out-of-bounds, OOB, Supplementary Figure 5), and found that model performance was significantly worse when compared to the source test datasets. The OOB testing performance is also significantly worse when compared to *Limit* and *Wide* testing (*results and discussion section 1*), which also contained peptides in the OOB range. Interestingly, the models performed slightly worse on heavier OOB peptides than on lighter OOB peptides (Figure 8), despite lighter peptides exhibiting higher individual variability, and the distribution of lighter peptides being more concentrated at one end of the distribution compared to the heavier peptides (Supplementary Figure 6). Similarly to Figure 6, we observe significantly more model uncertainty tied to earlier and later eluted peptides.

The significant reduction in performance we observed in the OOB testing suggests that the models do not learn the underlying nature of AA weights, folding, and physico-chemical properties, factors which

impact RT as well as the ionization and detectability of peptides, as much as they memorize the retention times of certain peptide sequence patterns [15].

Figure 8. Bayesian model uncertainty estimates for out-of-bounds peptides. For *PT17* (A), and *PT19* (B) we conducted model inference 25 times with layer dropouts applied at original rates. Each data point is an average of the inference results, with the colorization of the dots indicating the normalized relative variability of each peptide. For each of the datasets, we overlaid a distribution plot of the dataset retention time values as a solid line, and a linearity assessment (y=x) to facilitate a comparison of the model predictions with perfect alignment as a dotted line.

4 Fragmentation patterns

We also investigated the variability and content of fragmentation spectra in public data. In this case, as fragmentation does not influence peptide retention time, we will only consider the theoretical impact on deep learning applications like fragmentation pattern predictions and rescoring.

A perfect peptide fragmentation spectrum is a theoretical concept that consists of a discrete set of all characteristic peaks defined only by the peptide sequence. In reality, fragmentation spectra only contain subsets of these theoretical peaks with patterns based on the background contaminants from the instrument, fragmentation technique, collision energy, and more. In order to understand the challenges and limitations of the MS2 spectra for machine learning algorithms, we analyzed MS2 spectra peaks from 86 randomly sampled raw files containing more than 768 million peaks, allowing us to visualize the peak distributions found in MS2 spectra.

When looking at the distribution of all m/z values found in the 86 randomly sampled files, we observe a clear bimodal distribution independent of peak selection or bin sizes; one distribution is located at ~50-250 m/z, and the second distribution at ~250-2000 m/z. The distribution at the lower m/z range disrupts the expected normally distributed peptide fragment ions found at 250-2000 m/z, and consists of clearly distinguishable high-density peaks corresponding almost exclusively to single amino acid residues (Fig. 9E and 9F) and some background noise. The cumulative distribution plots in Figure 9 show the singly charged amino acid residues, where amino acids are annotated if the m/z of the peak matches the collision

ions *a*, *b*, or *y* or the electron-transfer ions *c* or *z*. The most abundant amino acid peaks are highlighted in Supplementary Table 1, and we observe that these peaks become more frequent at higher levels of peak selection (Supplementary Figures 7-8). It should be noted that the exact AA contribution to some peaks is uncertain, as multiple AA ions match the same m/z peaks.

Figure 9. **Illustration of how peak picking and peak binning affect the MS2 peak density plot and single amino acid density.** The density distributions of peak m/z values shown were obtained without peak picking (C, D) and with the top 50 peaks (A, B). The spectra were then imposed into 50 (A, C) and 500 (B, D) bins. For each value of peak picking, we also illustrated the cumulative distribution plot of the peaks in 50-250 m/z with single AA residues overlaid. No stratification on the fragmentation method or its settings has been considered during this analysis.

One thing not taken into consideration in this analysis is the method and energies used during fragmentation. The fragmentation technique, such as collision-induced dissociation (CID), high-energy C-trap dissociation (HCD), or electron-transfer dissociation (ETD), has a significant impact in determining the patterns of peptide fragmentations. This is due to each technique applying varying amounts and types of energy to the precursor ions, leading to distinct bond cleavage pathways and producing specific fragment ions [11]. Along with the fragmentation technique, it is also essential to discuss the fragmentation energy, often called normalized collision energy (NCE) for CID and HCD, and charge state-dependent reaction time for ETD. The stability of the created fragment ions depends primarily on the size, polarity, and charge on either side of the cleavage, and the energy with which the fragmentation occurs determines which bonds can be broken [20,21]. This also means that higher energy fragmentations are more likely to create single AA ions [22,23]. Utilizing the fragmentation-specific ion patterns, either by inclusion or stratification, has already been proven successful for both top-down and bottom-up predictive proteomics [10,11,24,25]. Due to the complexity of LC-MS/MS fragmentation patterns across fragmentation techniques and energy, some projects utilize project-specific peptide libraries to increase the identification of desired peptides. Even though the usage of peptide libraries will not directly affect the variability of peptide patterns in LC-MS or LC-MS/MS, they can still affect identified variability by having a significantly higher probability of identifying peptides in the library, while not identifying a

large number of otherwise present peptides. Recent developments in DL methods have enabled enhanced or library-free peptide identifications which, with future improvements, could increase efficacy without biasing the identification [26,27].

Conclusions.

Mass spectrometry remains a powerful tool to quantify thousands of protein abundances in biological samples. Analysis of the raw experimental data is increasingly dependent on suitable computational methods [28] with a major focus on algorithms for peptide identifications and protein quantifications. However, despite a variety of different statistical, conceptual, and graph approaches, methods such as database search engines still suffer from limitations both in accuracy and run time. Novel machine learning methods hold the promise of advancing the analysis of upcoming data, as well as having a high potential for re-purposing the ample body of public data for the retrieval of valuable new biological information.

In this manuscript, we have investigated and highlighted some of the main sources of variability found within the high-throughput MS data. We identified a range of factors that increase variability in the data-generating process and analyzed the homogeneity of the variability within a project when comparing different projects. Our main finding from the statistical analyses was that global variability, which is found between projects, is significantly larger than internal variability, which is found between files in the same projects. This is exhibited through instrument settings, sample preparation, and experimental choices, all of which are significantly more homogenous within any given project, compared between projects. Furthermore, we also wanted to see how these sources of variability impacted ML capabilities by training Prosit retention time predictors on each source individually, whenever applicable.

We trained nine Prosit models, tested these models on twenty-seven datasets, and performed transfer learning fourteen times. Our findings show that training models on data most closely resembling real-life test cases are crucial, as the models' ability to generalize outside the training data confinements are

severely limited. This is illustrated by the *PT* models outperforming any other model during training and validation while having considerable performance drops when tested on randomly acquired data, or peptides, not in the original m/z range.

Our results also found evidence that transfer learning can occasionally improve the performance of a pre-trained model. However, the most common scenario we observed was that models ended up mimicking non-transferred models for the same dataset, while not reducing the average amount of epochs needed for convergence. This tendency resulted in model regression in 5 of the 14 cases, and only resulted in model improvement in 1 of 14 cases. While our findings do indicate that models need to be trained on datasets from representative sources, they do not indicate that transfer learning outperforms training a new model in accuracy or computational needs (Supplementary Tables 4,5).

We argue, since a representative dataset is needed, that a research environment either has to train specialized models to their individual data collection methods or generate an unbiased dataset from publicly available data sources that attempts to mimic the intended post-training application through software such as MS2AI [18].

We further found that fragmentation spectra are rich in yet neglected information. Given the rather abundant single residue fragment ions, particularly present at higher activation energies, considerable amounts of internal ions should be present. This information has been so far mostly untapped due to the complexity to include internal ions in database search and spectrum prediction. Advanced machine learning methods might be capable of making sense of these ions despite their noisy and ubiquitous nature.

We note that a prevailing issue with the current data repositories is the missing or mislabeling of metadata. With the ongoing standardization efforts in large repositories such as PRIDE [29], this issue should fade over time. Through the analysis, we also identified the need to report more details about the experimental design, the data acquisition, and the post-processing in a comprehensive and standardized way to make them amenable as additional input for machine learning applications and thus allow for the direct training of the confounding factors.

Methods

We use different methods to evaluate the variability caused by different setup parameters of the LC-MS experiments and their effect on ML transferability to unseen data. To assess the impact of biases and experimental heterogeneity, we trained identical deep-learning models over a range of data properties and compared their results. We used the Prosit retention time model with peptide sequence and retention time as input and output, respectively. The Prosit model architecture consists of a sequence embedding layer, a bidirectional GRU layer, and an attention layer, followed by fully connected dense layers. The retention time of all peptides in a raw file has been linearly normalized to an effective gradient; spanning between the first (0) and last (1) identified peptide to mimic the iRT calculations performed in the original paper. No further data refinement or re-annotation has been applied to the files. Initially, we followed the hyperparameter setup described in the Prosit paper but found that 32 epochs were not sufficient for model training convergence. As a result, we increased the training to 100 epochs and applied a 20-epoch patience for early stopping instead. All other parameters were identical to those described in the original paper [11]. The Prosit deep learning architecture was implemented by using the DLOmix framework [30], and all modified peptides were removed due to DLOmix constraints.

For all trained networks we sample 10% of each dataset as a hold-out test set on which all testing is conducted. The remaining data is split into training and validation sets with a ratio of 80:20. For datasets composed of multiple PRIDE projects, the hold-out datasets consist of separate projects that were randomly sampled, whereas for datasets consisting of a single project, the hold-out dataset consists of randomly sampled raw files. This provides the most accurate heterogeneous test scenarios without overlap across projects or MS runs. Furthermore, the training and validation datasets are split by peptide sequences, meaning that no peptide will be present in both the training or the validation datasets. However, since the testing data is randomly split at the project or file level, these may contain sequences that are also present in the training datasets.

The data acquisition along with filtering and model training and testing was managed using MS2AI with MongoDB in Python 3.8 with an NVIDIA v100 GPU. The data were acquired in November 2021 with the extractor API with the options “-p -mo -t 128” which allows for en-mass data acquisition from PRIDE (-p) while only fetching MaxQuant information (-mo), and increasing thread counts to 128 to allow for faster runtime (-t 128). This requires the current version of the PRIDE metadata which is downloaded using the scraper API and the -db option. The filtering was performed using the filter API using the -q option with MongoDB or string filters available in the GitHub repository. The model training and testing were performed using the network API with “-t prosit -e 100 -sos -s *n*” to train a Prosit model for 100 epochs with training and validation being split based on unique sequences and a given seed for consistent training, validation, and test splitting. When performing transfer learning, the only difference is “-t prosit-ID” which uses the weights of the trained model with the same ID. Model training times varied from 4 to 10 hours based on the size of the dataset and epochs needed to converge. All code is available in the GitHub repository along with the seeds for the runs.

We utilized a Bayesian approximation of the model uncertainty, by performing model inference with dropout enabled [31,32]. The real retention time values are then plotted against the mean predicted values, with the color of the data point corresponding to the normalized variances of the predicted values. The dropout for inference testing was applied to all layers where dropout was originally applied, with original dropout rates. This allows us to evaluate the models not just on their metric performances, but also determine the retention time ranges where the models are least certain of their predictions. This method is available in MS2AI network API using the “-id *n*” option to run *n* dropout tests and automatically generate the data visualization plots.

1 Single vs multi-project variability

In order to measure the difference in variability not caused by individual factors, but instead caused by systemic changes in experimental protocol, we compared the model performance of two single-project

models to the performance of two multi-project models. We did this by training four Prosit models on data from four different sources: 2017 [33] and 2019 [34] ProteomeTools datasets (*PT17* and *PT19*, respectively) and two sets of acquired data from randomly sampled PRIDE projects; one limited to the 750.000 peptides filtered at 100 Andromeda score threshold, which is the score reported by MaxQuant (*Limit*), and one with 2.000.000 peptides filtered at 150 Andromeda score threshold (*Wide*). Alongside the initial training and testing, we also performed transfer learning on all models for all non-source datasets to compare their initial performance to post-transfer learning performance.

2 Spectra and gradient lengths

To compare and analyze the variability in gradient lengths, we extracted the metadata from each raw file (found in the *files.bson.gz*) and we extracted the gradient lengths of all runs individually, which we plotted in a histogram against the probability of each gradient length. We then calculated the cumulative distribution function of the gradient lengths for all files which we overlaid on the histogram. Then we calculated the number of unique gradients across all files from the same PRIDE accession, allowing us to visualize the variability found within projects when plotting the number of unique gradients in a histogram against the probability of each number of unique gradients.

Along with gradient length visualizations, we also trained two Prosit models to evaluate the effect of gradient length on model performance. The models were trained datasets that were divided into two groups based on their gradient lengths: short (≤ 60-minute gradients) and long (> 100-minutes gradients). The data were randomly sampled from the full 398M peptide dataset and only peptides with ≥ 150 Andromeda scores were kept. We also performed transfer learning of both gradient models to the opposing datasets. Furthermore, to test model uncertainty across the gradient, we performed model inference with dropout enabled on all four models, as described in method section 1.

3 m/z range filter

To visualize and compare m/z filters across files and projects, we extracted the m/z filter bounds from each raw file, and plotted the lower bounds, upper bounds and the difference between the upper and lower bounds to get the lengths. We then visualized these values in a violin plot, in order to see possible patterns or key values in the distributions.

In order to evaluate the impact of m/z filters on model performance we created two subsets of data, this time much smaller due to low peptide count; one in which all peptides lie below 360 m/z (*Lower*), and one in which all peptides lie above >1300 m/z (*Upper)*. These bounds were chosen as we are going to re-use the *PT17* and *PT19* models, which have m/z filter bounds of 360-1300 m/z, and using these datasets will allow us to evaluate peptides that are outside original filter bounds. As we did not train new models for this section, no transfer learning was applied. Again we also perform model inference with dropout enabled, in order to assess the model uncertainty for these out-of-bounds (OOB) peptides.

4 Fragmentation Pattern

To analyze the distribution of MS2 peaks we extracted every peak from 86 raw files [35], where we compared the raw spectra with all peaks preserved, to spectra filtered by top *n* peaks based on intensity for three values of *n*: 50, 100, and 200. MS2 spectra are often annotated or binned to make them fit into typical ML architectures. To illustrate how this type of binning affects the outcome distribution, we also binned each of the combined peak selected spectra at 50, 100, 200, and 500 total bins from 0-2000 m/z. We then calculated the collision ions *a*, *b*, and *y* as well as the ETD ions *c*, and *z* for all amino acids separately. This was done by adding -27, 1, 19, 18, and 2 mass to their single charged molecular residue weights for *a*, *b*, *y*, *c*, and *z* ions respectively [36,37].

Abbreviations

ML: Machine Learning

DL: Deep Learning

MS: Mass Spectrometry

LC-MS or MS1: Liquid Chromatography-Mass Spectrometry

LC-MS/MS, MS/MS or MS2: Tandem mass-spectrometry

m/z: Mass to charge ratio

NCE: Normalized Collision Energy

PTM: Post-translational modification

CID: Collision induced dissociation

HCD: high-energy C-trap dissociation

ETD: electron-transfer dissociation

ETciD: electron-transfer and collision-induced dissociation

EThcD: electron-transfer and higher-energy collision dissociation

PX: ProteomeXchange

References

1. Aebersold R, Mann M. Mass-spectrometric exploration of proteome structure and function. *Nature*. 537:347–552016;

2. Altelaar AFM, Munoz J, Heck AJR. Next-generation proteomics: towards an integrative view of proteome dynamics. *Nat Rev Genet*. 14:35–482013;

3. Noor Z, Ahn SB, Baker MS, Ranganathan S, Mohamedali A. Mass spectrometry–based protein identification in proteomics—a review. *Brief Bioinform*. Oxford Academic; 22:1620–382020;

4. Bantscheff M, Schirle M, Sweetman G, Rick J, Kuster B. Quantitative mass spectrometry in proteomics: a critical review. *Anal Bioanal Chem*. 389:1017–312007;

5. Yadav A, Marini F, Cuomo A, Bonaldi T. Software Options for the Analysis of MS-Proteomic Data. *Methods Mol Biol*. 2361:35–592021;

6. Deutsch EW, Csordas A, Sun Z, Jarnuczak A, Perez-Riverol Y, Ternent T, et al.. The ProteomeXchange consortium in 2017: supporting the cultural change in proteomics public data deposition. *Nucleic Acids Research*. 45:D1100–62017;

7. Rehfeldt TG, Gabriels R, Bouwmeester R, Gessulat S, Neely BA, Palmblad M, et al.. ProteomicsML: An Online Platform for Community-Curated Data sets and Tutorials for Machine Learning in Proteomics. *J Proteome Res*. :632–62023;

8. Moruz L, Käll L. Peptide retention time prediction. *Mass Spectrom Rev*. 36:615–232017;

9. Bonini P, Kind T, Tsugawa H, Barupal DK, Fiehn O. Retip: Retention Time Prediction for Compound Annotation in Untargeted Metabolomics. *Anal Chem*. 92:7515–222020;

10. Wen B, Zeng W-F, Liao Y, Shi Z, Savage SR, Jiang W, et al.. Deep Learning in Proteomics. *Proteomics*. 20:e19003352020;

11. Gessulat S, Schmidt T, Zolg DP, Samaras P, Schnatbaum K, Zerweck J, et al.. Prosit: proteome-wide prediction of peptide tandem mass spectra by deep learning. *Nat Methods*. 16:509–182019;

12. Ma C, Ren Y, Yang J, Ren Z, Yang H, Liu S. Improved Peptide Retention Time Prediction in Liquid Chromatography through Deep Learning. *Anal Chem*. 90:10881–82018;

13. Moruz L, Staes A, Foster JM, Hatzou M, Timmerman E, Martens L, et al.. Chromatographic retention time prediction for posttranslationally modified peptides. *Proteomics*. 12:1151–92012;

14. Degroeve S, Martens L. MS2PIP: a tool for MS/MS peak intensity prediction. *Bioinformatics*. 29:3199–2032013;

15. Naim Abdul-Khalek, Reinhard Wimmer, Michael Toft Overgaard, Simon Gregersen Echers. Insight on physicochemical properties governing peptide MS1 response in HPLC-ESI-MS/MS: A deep learning approach. *Comput Struct Biotechnol J*. Elsevier; 21:3715–272023;

16. Meier F, Köhler ND, Brunner A-D, Wanka J-MH, Voytik E, Strauss MT, et al.. Deep learning the collisional cross sections of the peptide universe from a million experimental values. *Nat Commun*. 12:11852021;

17. Cox J, Mann M. MaxQuant enables high peptide identification rates, individualized p.p.b.-range mass accuracies and proteome-wide protein quantification. *Nat Biotechnol*. 26:1367–722008;

18. Rehfeldt TG, Krawczyk K, Bøgebjerg M, Schwämmle V, Röttger R. MS2AI: Automated repurposing of public peptide LC-MS data for machine learning applications. *Proteomics*. 38:875–72022;

19. Rose CM, Rush MJP, Riley NM, Merrill AE, Kwiecien NW, Holden DD, et al.. A calibration routine for efficient ETD in large-scale proteomics. *J Am Soc Mass Spectrom*. 26:1848–572015;

20. Mann M, Jensen ON. Proteomic analysis of post-translational modifications. *Nat Biotechnol*. Nature Publishing Group; 21:255–612003;

21. McLafferty FW, Turecek F. Interpretation Of Mass Spectra. University Science Books;

22. Syka JE, Coon JJ, Schroeder MJ, Shabanowitz J, Hunt DF. Peptide and protein sequence analysis by electron transfer dissociation mass spectrometry. *Proc Natl Acad Sci U S A*. Proc Natl Acad Sci U S A; 2004; doi: 10.1073/pnas.0402700101.

23. Olsen JV, Macek B, Lange O, Makarov A, Horning S, Mann M. Higher-energy C-trap dissociation for peptide modification analysis. *Nat Methods*. Nat Methods; 2007; doi: 10.1038/nmeth1060.

24. Liu K, Li S, Wang L, Ye Y, Tang H. Full-Spectrum Prediction of Peptides Tandem Mass Spectra using Deep Neural Network. *Anal Chem*. 92:4275–832020;

25. Guan S, Moran MF, Ma B. Prediction of LC-MS/MS Properties of Peptides from Sequence by Deep Learning. *Mol Cell Proteomics*. 18:2099–1072019;

26. Sinitcyn P, Hamzeiy H, Salinas Soto F, Itzhak D, McCarthy F, Wichmann C, et al.. MaxDIA enables library-based and library-free data-independent acquisition proteomics. *Nat Biotechnol*. Nature Publishing Group; 39:1563–732021;

27. Degroeve S, Gabriels R, Velghe K, Bouwmeester R, Tichshenko N, Martens L. ionbot: a novel, innovative and sensitive machine learning approach to LC-MS/MS peptide identification.

28. Tsiamis V, Ienasescu H-I, Gabrielaitis D, Palmblad M, Schwämmle V, Ison J. One Thousand and One Software for Proteomics: Tales of the Toolmakers of Science. *J Proteome Res*. 18:3580–52019;

29. Perez-Riverol Y, Alpi E, Wang R, Hermjakob H, Vizcaíno JA. Making proteomics data accessible and reusable: current state of proteomics databases and repositories. *Proteomics*. 15:930–492015;

30. wilhelm-lab: GitHub - wilhelm-lab/dlomix: Python framework for Deep Learning in Proteomics. GitHub. https://github.com/wilhelm-lab/dlomix Accessed 2022 Mar 2.

31. Gal Y, Ghahramani Z. Dropout as a Bayesian Approximation: Representing Model Uncertainty in Deep Learning. In: Balcan MF, Weinberger KQ, editors. *Proceedings of The 33rd International Conference on Machine Learning*. New York, New York, USA: PMLR; p. 1050–9.

32. Neely BA, Dorfer V, Martens L, Bludau I, Bouwmeester R, Degroeve S, et al.. Toward an Integrated Machine Learning Model of a Proteomics Experiment. *J Proteome Res*. 22:681–962023;

33. Daniel P Zolg, Mathias Wilhelm, Karsten Schnatbaum, Johannes Zerweck, Tobias Knaute, Bernard Delanghe, Derek J Bailey, Siegfried Gessulat, Hans-Christian Ehrlich, Maximilian Weininger, Peng Yu, Judith Schlegl, Karl Kramer, Tobias Schmidt, Ulrike Kusebauch, Eric W Deutsch, Ruedi Aebersold, Robert L Moritz, Holger Wenschuh, Thomas Moehring, Stephan Aiche, Andreas Huhmer, Ulf Reimer & Bernhard Kuster. Building ProteomeTools Based on a Complete Synthetic Human Proteome. *Nature Methods*. 14:259–622017;

34. Wang D, Eraslan B, Wieland T, Hallström B, Hopf T, Zolg DP, et al.. A deep proteome and transcriptome abundance atlas of 29 healthy human tissues. *Mol Syst Biol*. 15:e85032019;

35. Rehfeldt T. data reference https://doi.org/10.6084/m9.figshare.21680669.v2. figshare;

36. : amino acid residues molecular masses. Riken. http://www2.riken.jp/BiomolChar/Aminoacidmolecularmasses.htm Accessed 2023 Mar 20.

37. : PROTEOMICS TOOLKIT. http://db.systemsbiology.net/proteomicsToolkit/FragIonServlet.html Accessed 2023 Mar 20.

38. : Peptide Sets. http://www.proteometools.org/index.php?id=49 Accessed 2021 Jun 3.

Authors' contributions

T.G.R., K.K., R.R, V.S. conceptualized the study. T.G.R. designed and carried out the analyses with the help of K.K. T.G.R. wrote the first draft of the manuscript, with all authors contributing to the revisions. All authors read and approved the final manuscript.

Acknowledgments

Funding

This work was supported by the Velux Foundation [00028116].

Availability of source code and requirements

Project name: MS Review Paper

Project home page: https://gitlab.com/tjobbertjob/ms-review-paper

Operating system(s): Platform independent

Programming language: Python

Other requirements: Python 3.8, MongoDB

License: e.g. GNU AGPLv3

Data Availability

The database files, reference texts, and trained models supporting the results of this article are available in the FigShare repository [35].

Competing interests

The authors declare that they have no competing interests.

Author information

University of Southern Denmark, Department of Mathematics and Computer Science. Campusvej 55, 5230 Odense
Tobias Greisager Rehfeldt, Konrad Krawczyk & Richard Röttger

Aalborg University, Department of Chemistry and Bioscience. Fredrik Bajers Vej 7H, 9220 Aalborg
Simon Gregersen Echers

Technical University of Denmark, Department of Health Technology. Ørsteds Pl. 345C, 2800 Kongens Lyngby
Paolo Marcatili

University of Southern Denmark, Department of Biochemistry and molecular biology. Campusvej 55, 5230 Odense
Pawel Palczynski & Veit Schwämmle

Corresponding Author: Veit Schvämmle

Figure 1 Click here to access/download;Figure;Figure 1.png

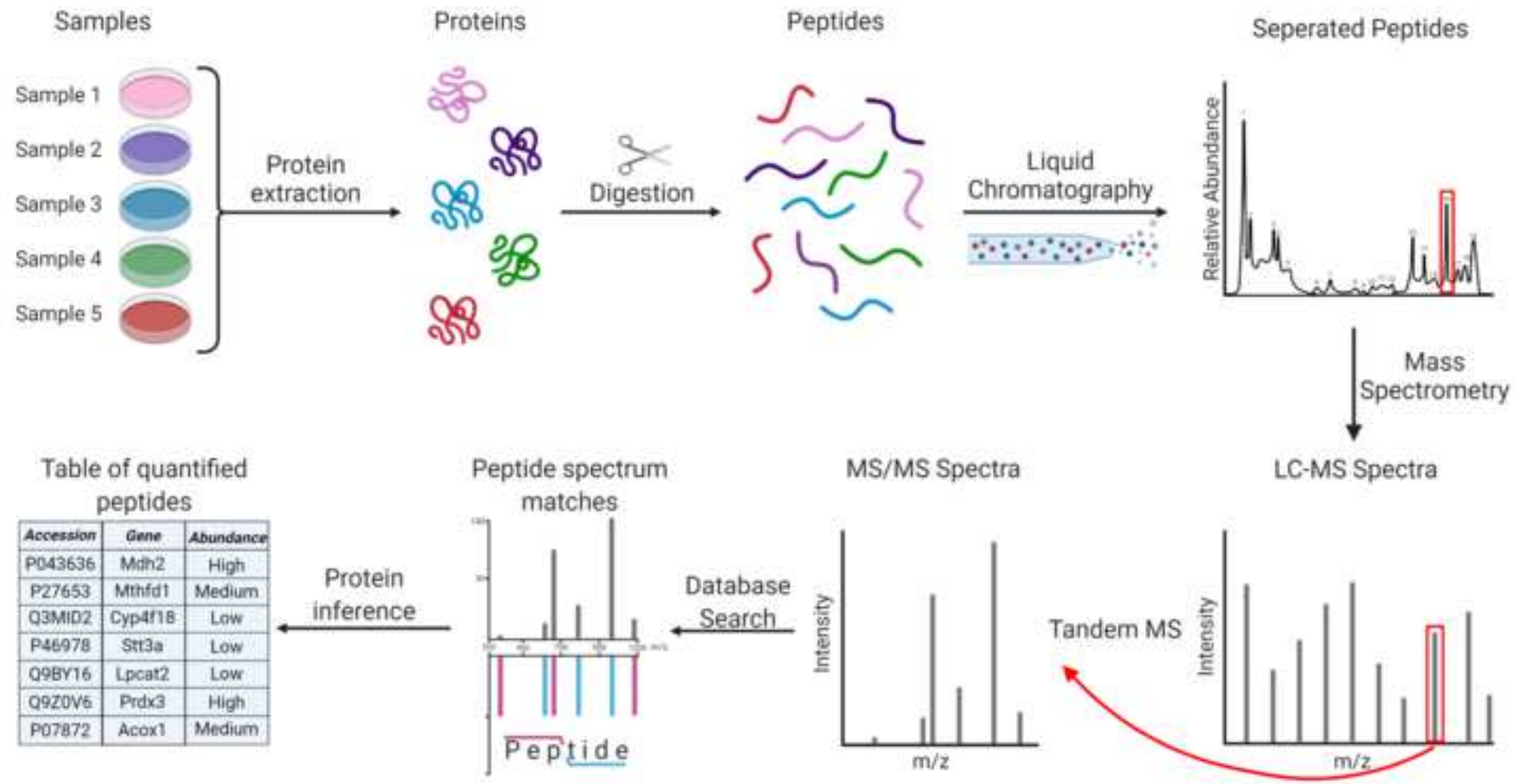

Figure 2

Click here to access/download;Figure;Figure 2.png

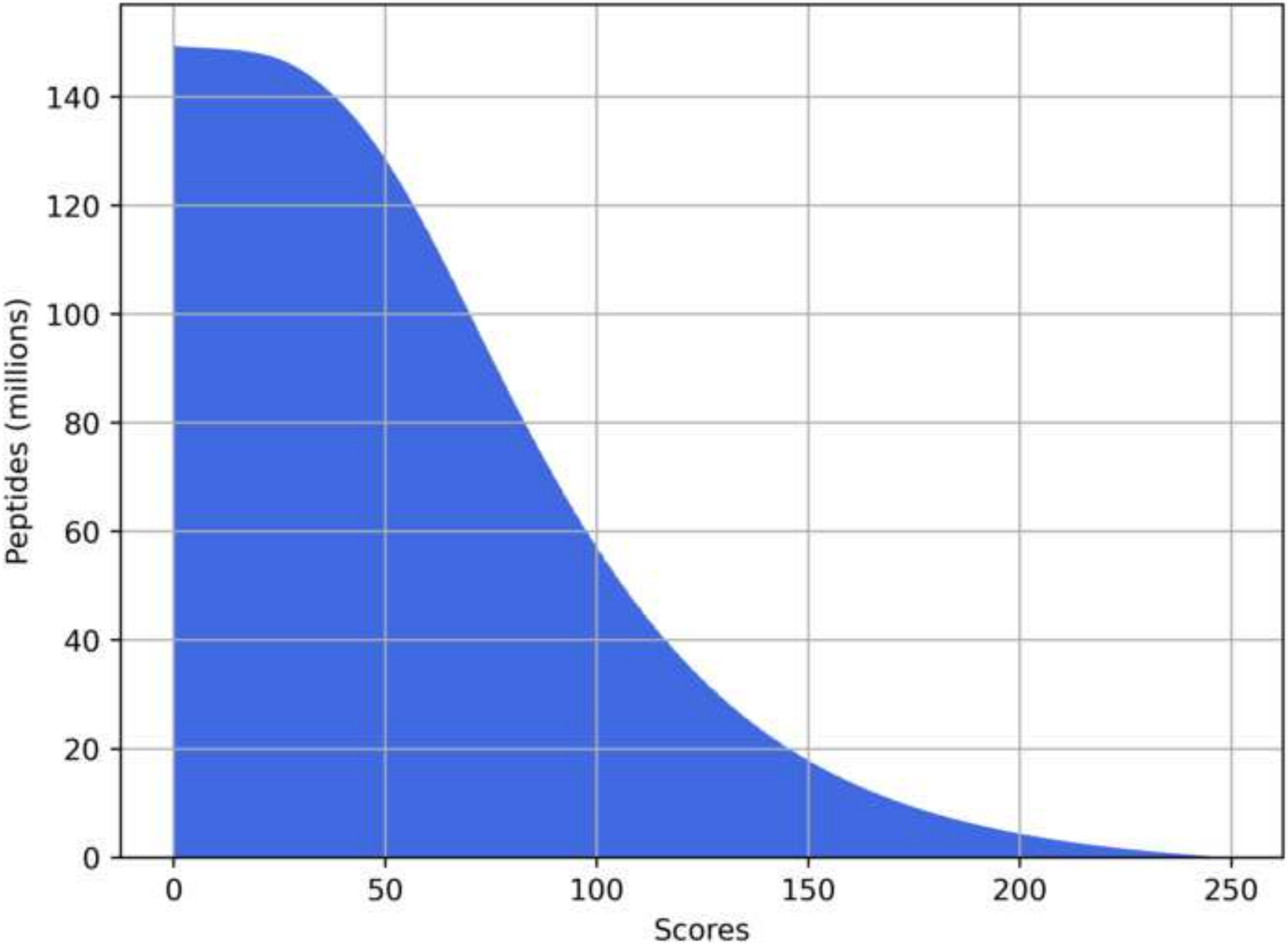

Figure 3

Click here to access/download;Figure;Figure 3.png

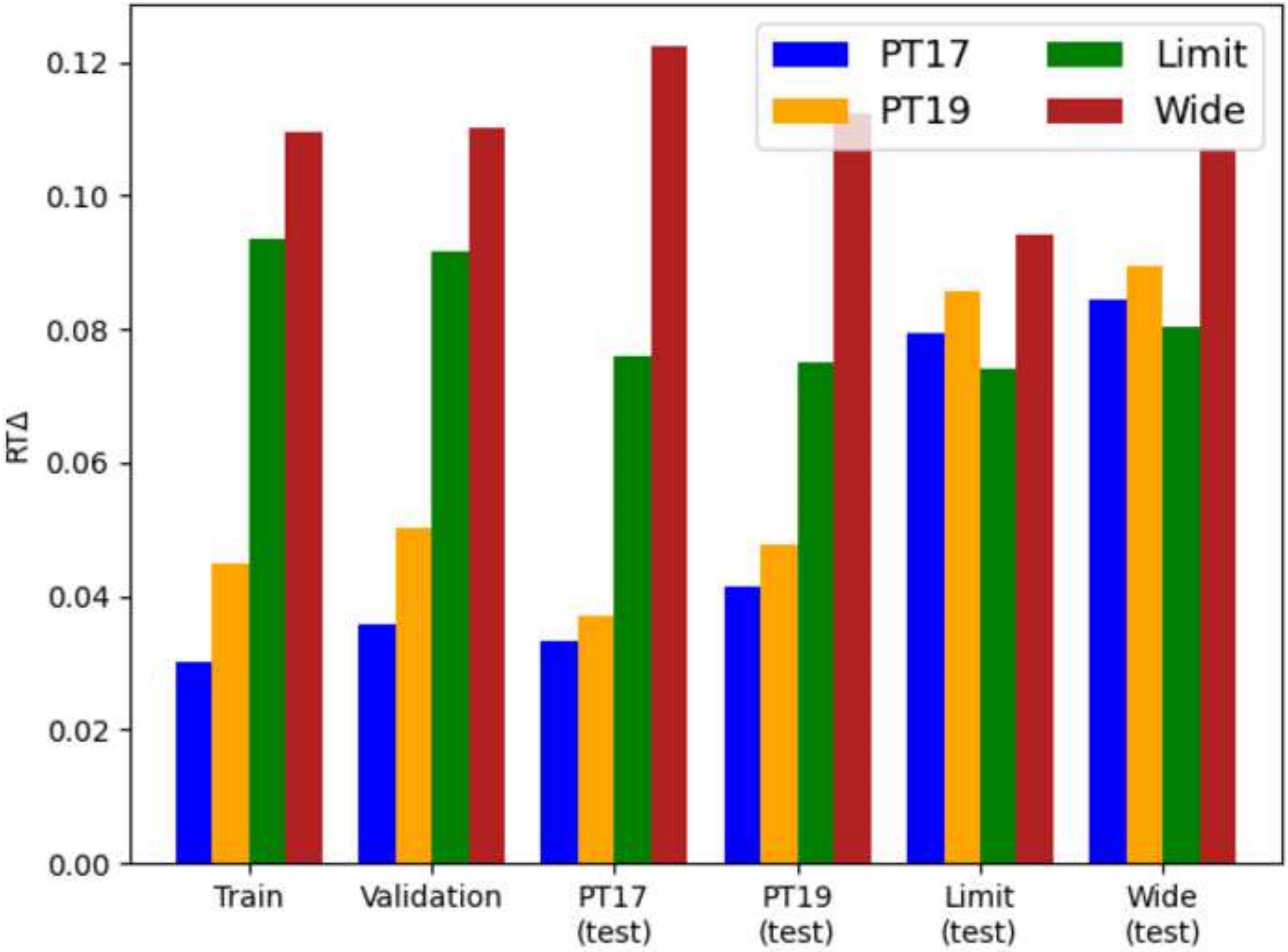

Figure 4

Click here to access/download;Figure;Figure 4.png

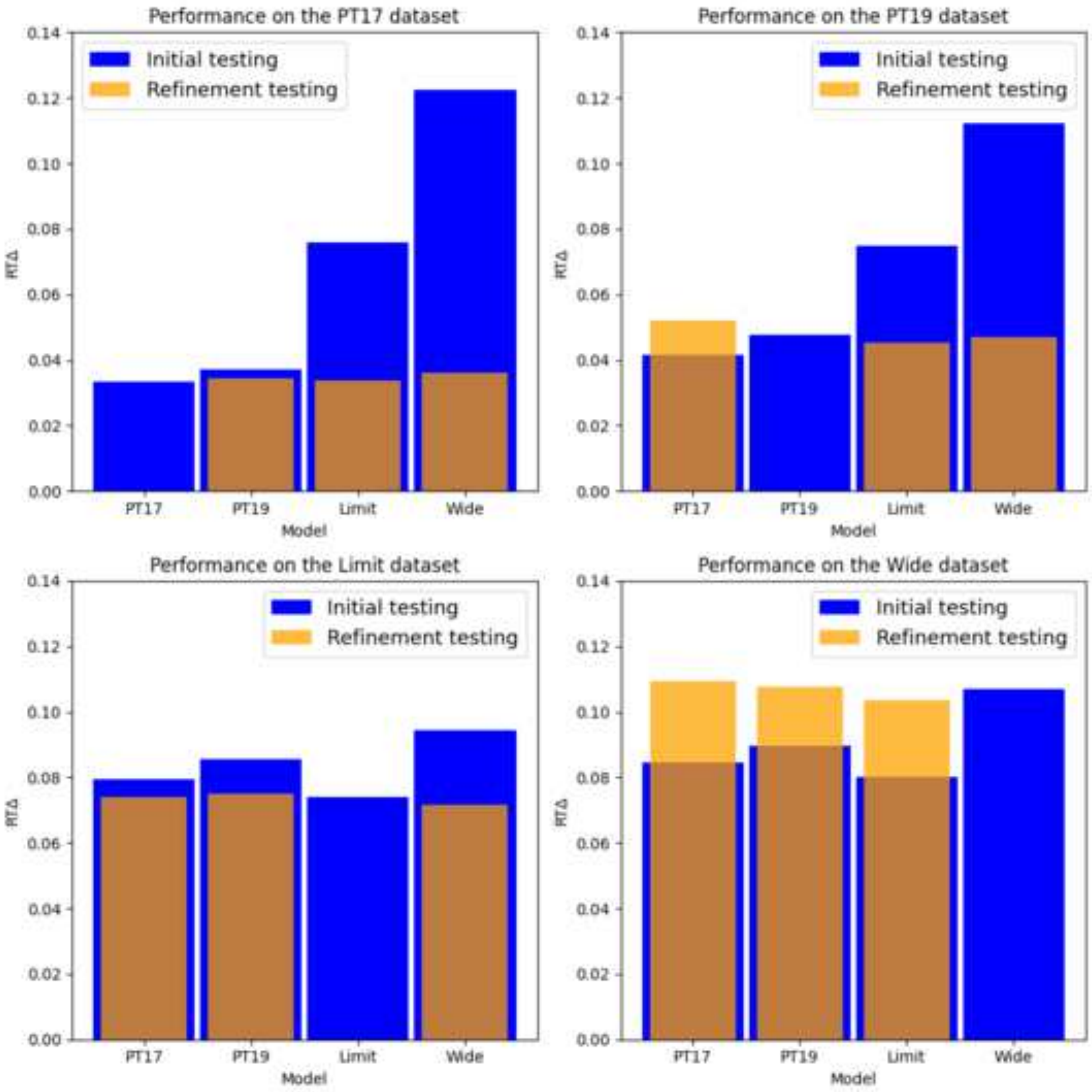

Figure 5

Click here to access/download;Figure;Figure 5.png

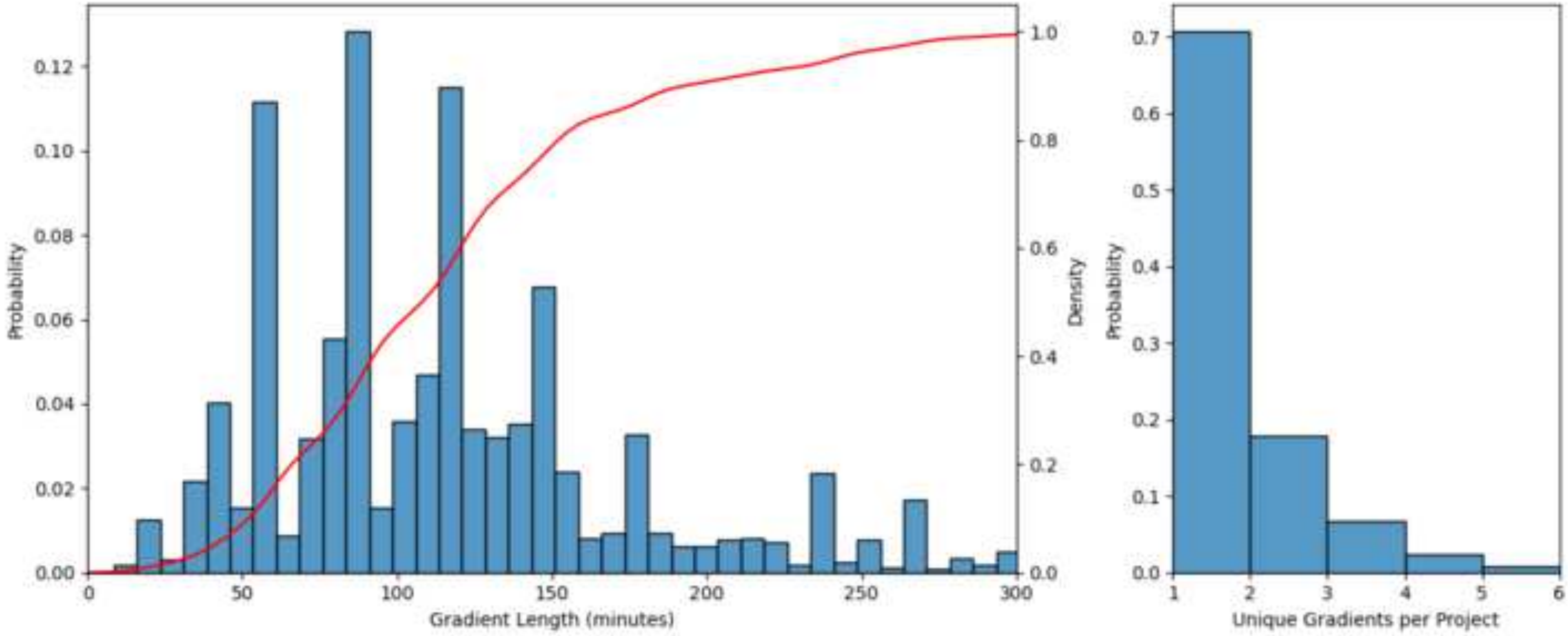

Figure 6

Click here to access/download;Figure;Figure 6.png

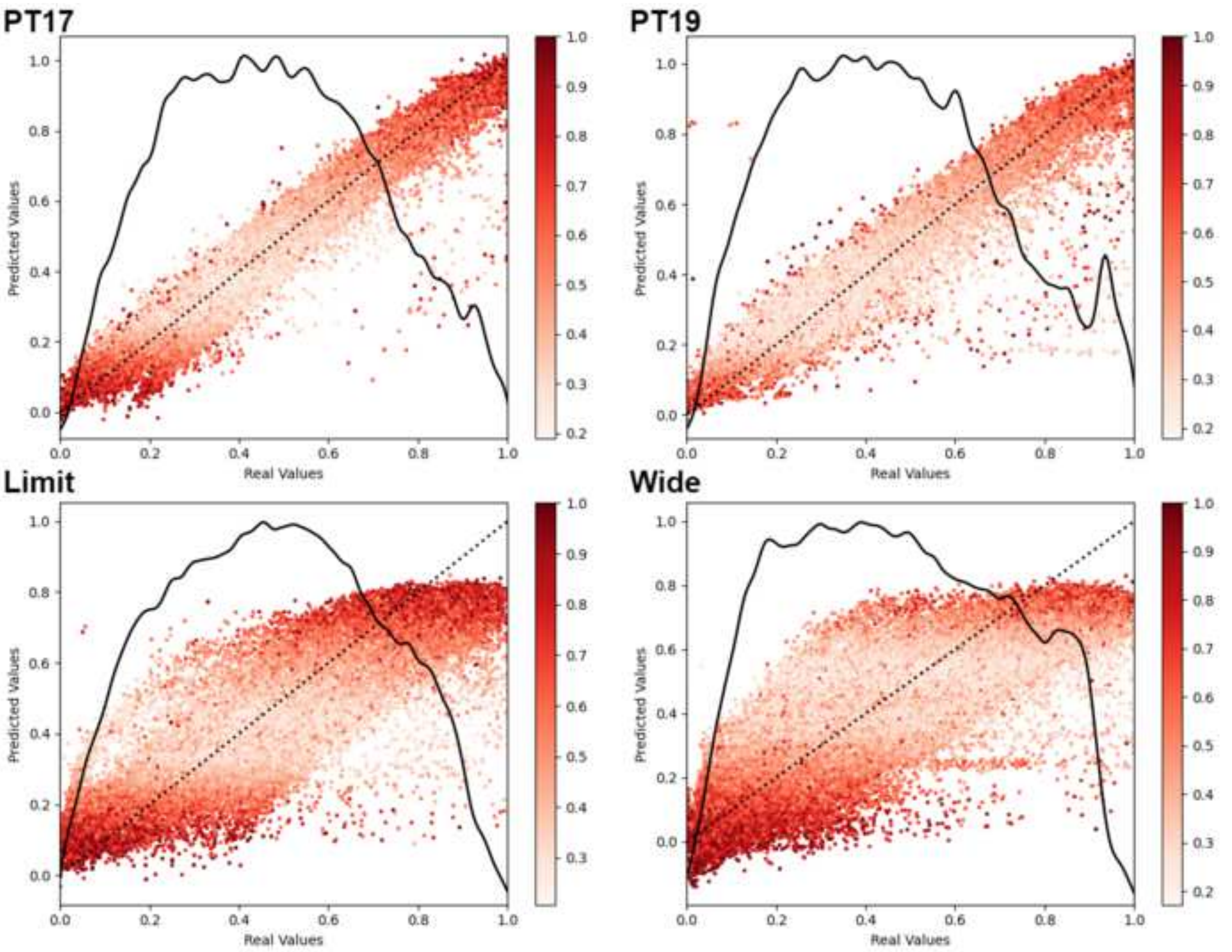

Figure 7

Click here to access/download;Figure;Figure 7.png

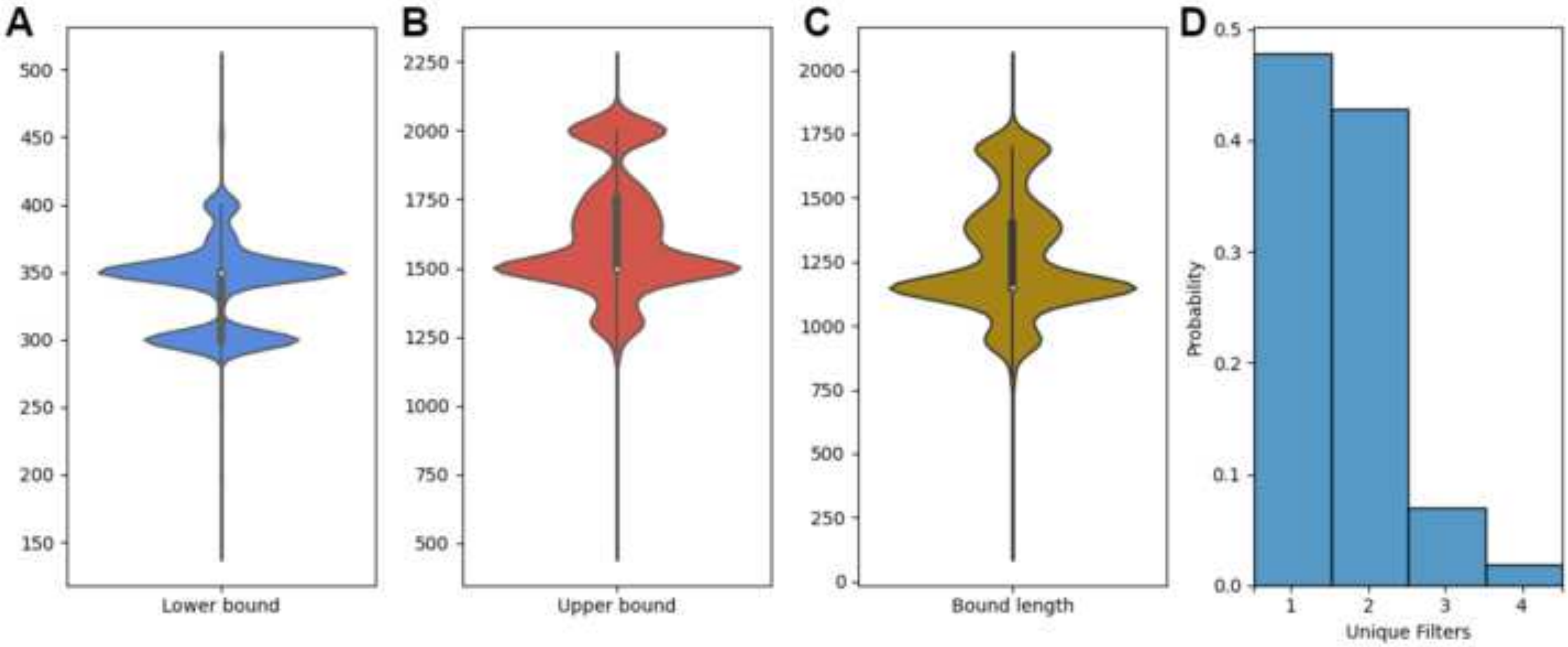

Figure 8

Click here to access/download;Figure;Figure 8.png

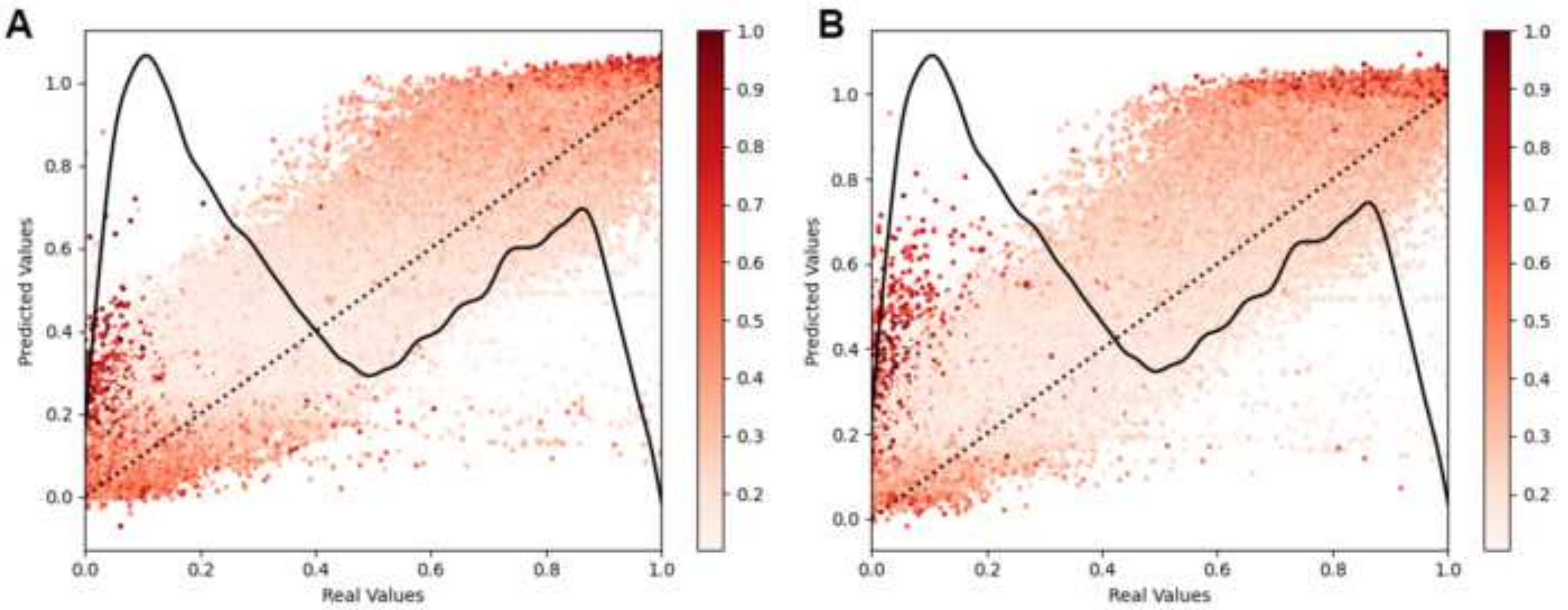

Figure 9

Click here to access/download;Figure;Figure 9.png

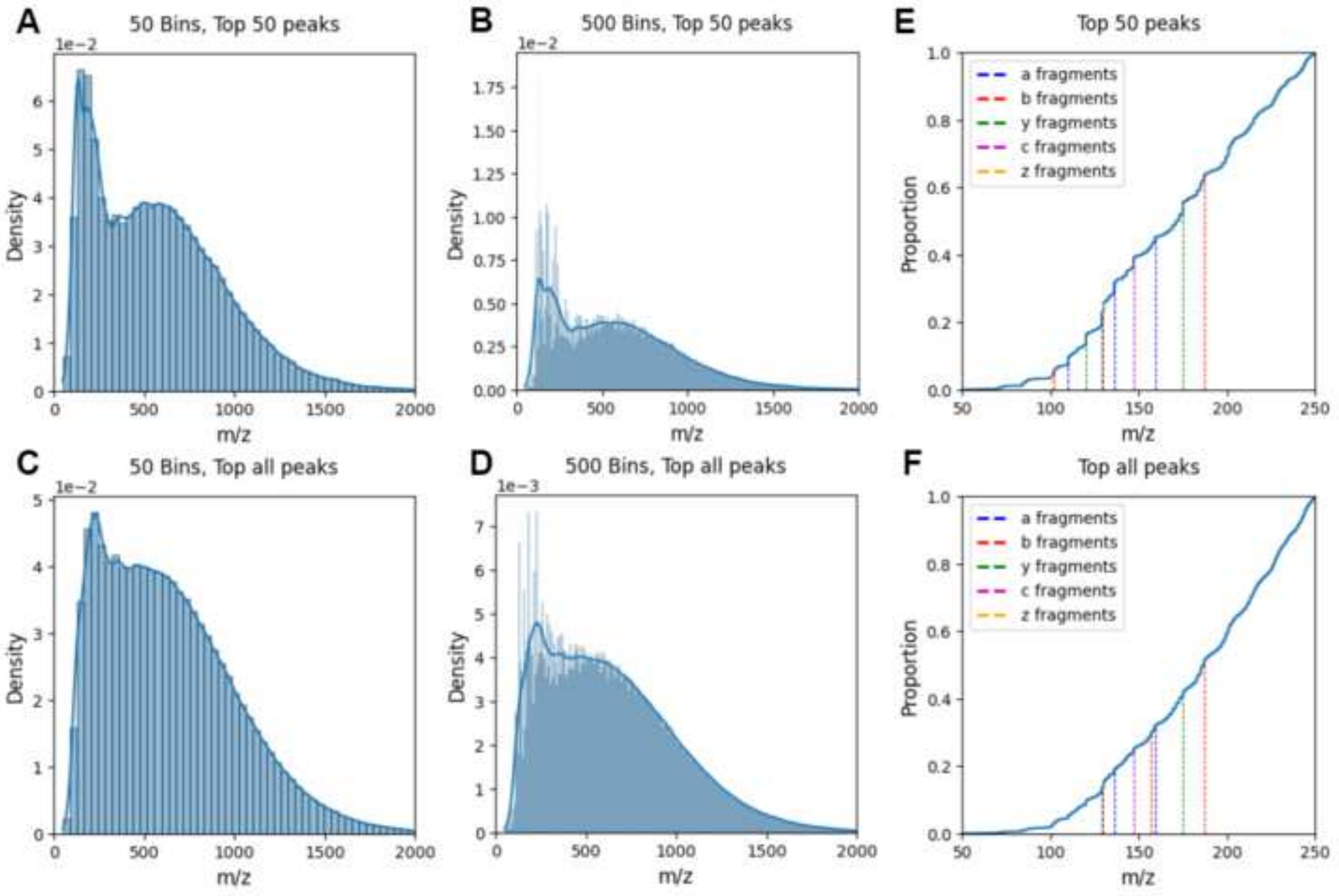

Click here to access/download
Supplementary Material
DOME registry file for -094.png

Click here to access/download
Supplementary Material
Evaluation metric report.pdf

Supplementary figures

Click here to access/download
Supplementary Material
Supplementary Material.pdf

Manuscript with tracked changes

Click here to access/download
Supplementary Material
ManuscriptTrackedChanges.pdf
