# Supplementary material for: Variability analysis of LC-MS experimental factors and their impact on machine learning
Source: Gigascience. 2023 Nov 20;12:giad096. doi: 10.1093/gigascience/giad096 (PMC10659119; doi:10.1093/gigascience/giad096)
Supplement: giad096_Response_to_Reviewer_Comments_Original_Submission [file giad096_response_to_reviewer_comments_original_submission.pdf]

# Author's Response To Reviewer Comments

-----------
We want to thank the reviewers for their excellent feedback, allowing us to improve the quality of the reports and writing in the paper.
-----------

Reviewer reports:
Reviewer #1: This paper aimed to facilitate machine learning efforts in mass spectrometry data by conducting a systematic analysis of the potential sources of variance in public mass spectrometry repositories. This paper examined how these factors affect machine learning performance and performed a comprehensive transfer learning to

Please explain the rationality of the RT used for evaluating model performance. In addition, it is necessary to increase other evaluation metrics to provide a more powerful comparison of model performance.

-----------
ΔRT is the metric used by the creators of the DLOmix package from the Lab which created the Prosit model, which is why we decided to use this metric. This explanation has also been added to the manuscript. However, during training, we also monitored MSE and MAE which we have added to the paper for further comparison. While MSE often showed more significant differences between training and testing, all models exhibited the same patterns regardless of the metric used for evaluation. We have added an explanation for the choice of a metric within the manuscript, as well as added an "Evaluation Metric Report.pdf" file which contains every model performance for both ΔRT, MSE, and MAE, illustrating performance invariance across metrics.
-----------

The curves in Figures 6 and 8 should provide more explanations to help readers understand. In addition, all figures are somewhat blurry and clearer figures should be provided.

-----------
We have updated the figure text for both figures 6 and 8 to help readers understand. The images we provided to GigaScience were not of low quality or DPI. We suspect that the quality loss was produced during the conversion of the uploaded files.
-----------

This paper does not provide specific implementation steps of variance. Please describe the variance analysis process in mathematical language and provide the corresponding mathematical formula.

-----------
We apologize for using a slightly misleading expression. We never intended to use "variance" in the mathematical sense but more as a surrogate or heuristic of variability found within the data. As complex LC-MS data has no meaningful way of computing mean or variance values we are incapable of providing single values for variance in any meaningful sense. Instead, we have replaced the word "variance" with "variability" throughout the manuscript.
-----------

There are some formatting issues: Keywords and the title 'Data Description' should only have the first letter capitalized. On pages 6, 17, and 18, the font size of the article is inconsistent.

-----------
Thank you for pointing this out. We fixed the capitalization of the Data description and certain keywords. The inconsistent font and font size were not present in the document we sent to GigaScience. Why the text was inconsistent when the reviewers received them, we do not know.
-----------

There are some grammar issues: On pages 6 and 16, dataset should be added with 's'. On page 7, lines 9-10, the tense is not unified.

-----------
We fixed a few cases of datasets either having or missing and ‘s’ at the end. We also fixed the tense issue on page 7
-----------

There are significant issues with the format of references. Inconsistent capitalization of initial letters in literature titles, such as [1] and [5]; Some literature lacks page numbers, such as [6] and [18]. Please re-organize the references according to the format required by the journal.

-----------
We have recognized errors in the references section and fixed some errors with capitalization and page numbers. However, according to the GigaScience submission rules, we should not apply Title Case to references and have altered all titles to be as they were initially published.
-----------

Reviewer #2: The paper applies machine learning to publicly available proteomics data sets and assesses the ability to transfer learning algorithms between projects. The primary aim of these algorithms appears to be an attempt to increase consistency of retention time prediction for data-dependent acquisition data sets, however this is not explicitly stated within the text. The application of machine learning to derive insight from previous performed proteomics experienced is a worthwhile exercise.

The authors report ΔRT to determine fitting for their models. It would be interesting to see whether the models had other metrics used to assess fitting, or could be used to increase number of identifications within sample sets, and whether this was successful. ALternatively, was there any conclusions able to be drawn about peptide structure and RT determination from these models?

-----------
We thank the reviewer for this important point. It was also brought up by reviewer 1, so we will append the same response here: ΔRT is the metric used by the creators of the DLOmix package from the Lab which created the Prosit model, which is why we decided to use this metric. However, during training, we also monitored MSE and MAE which we have added to the paper for further comparison. While MSE often showed more significant differences between training and testing, all models exhibited the same patterns regardless of the metric used for evaluation. We have added an explanation for the choice of a metric within the manuscript, as well as added an “Evaluation Metric Report.pdf” file which contains every model performance for both ΔRT, MSE, and MAE, illustrating performance invariance across metrics.
Due to constraints during the project and the extensive training of the models, we were, unfortunately, unable to do tests on peptide structure.
-----------

Project specific libraries are well known to improve results compared with publicly available databases, and the discussion on this point should be developed further through comparison of this work with other papers - particularly with advances in machine learning and neural networks in the data independent analysis field.

-----------
This is an important point. We have added a short section in the “fragmentation pattern” section where we discuss project specific library’s ability to improve peptide identifications while also highlighting how such libraries can bias the datasets. However, as our work does not consider the efficacy of peptide identifications, but rather the variability of the peptide elutions, we do not believe that it would merit a larger change of the manuscript.
-----------

Comparison of Q-Exactiv models vs Orbitraps appears to be somewhat redundant, and possible a result of

poor meta-data as Q-Exactiv instruments are orbitrap mass spectrometers. A more interesting comparison to make here would be between orbitrap and TOF instruments, though as the datasets have all been processed through MaxQuant, it is likely the vast majority were acquired on orbitrap instruments.

-----------
We agree with the reviewer on this point and have opted to remove the Q. Exactive vs Orbitrap model comparison from the manuscript as well as the supplementary material. However, during data extraction, we were unable to acquire an adequate amount of ToF data points to conduct any meaningful statistics. This results in almost all data being either orbitrap or Q. Exactive instruments, at a relatively equal split.
-----------

The paper uses ΔRT as the readout for all models tested, however the only chromatography variable considered in testing the models is gradient length. However, chromatography is also dependent on column chemistry, column dimensions, composition of buffer, use of traps, temperature etc. These are also likely to be contributing the variance observed between the PT datasets where these variables will be consistent and publicly available datasets. These factors are also likely to play a role in higher uncertainty for early and late eluting peptides where these factors are likely to vary most between sample sets. The metadata may not be available to use to compare within the data sets selected, so the authors should at minimum make discussion around these points.

-----------
We agree with the reviewer, that we lack a proper discussion of other variability-inducing factors in the LC-MS system setup. These factors are difficult to obtain and we thus
omitted investigating their impact. We have added new paragraphs which discuss these factors in detail, and why we are unable to train models or compute statistics with these factors.
-----------

Sample preparation is likely to have similar effects, and as the PT datasets are generated synthetically using ideal peptides, publicly available datasets will be generated from complex sample mixtures, and have increased variance due to inefficiencies of digestion, sample clean up and matrix effects. Previous studies on variance have also described sample preparation as the highest cause of variance. This needs further discussion

-----------
This point extends on the previous point, and we have extended the section on LC-MS system variability concerns to also encompass sample preparation. This section highlights the problem of unaccountable factors which are often under-reported and can not extracted from the raw files.
-----------

While the isolation windows of the m/z will lead to unobserved space, search engines setting will also apply here. From the text, it appears that the only spectra that were considered were those already identified in a search program (due to having Andromeda cut-off scores always apply). Typical setting for a database search will have a cut off of peptide sequences of at least 7 residues, making peptide masses appearing lower than 350 m/z unlikely. There is also significant amount of noise below 350 m/z and this also likely contributes to poorer fitting.

-----------
We agree with the reviewers' points here and have added a short paragraph to section 3 which mentions database search cut-offs and noise in the lower end of the m/z space.
-----------

The authors identify differences in MSMS spectral features, however, most of these points are well known in the field. The authors should develop the discussion on the causes of the differences in fragmentation, as CID low mass drop off is expected, and the change in profile is expected with increasing activation energies. A more developed analysis could exclude precursor masses from these plots and focus solely on fragment ions generated.

-----------

We agree that the fragmentation technique and energy section comprised too many confusing and misleading methodologies, and did not align well with the retention time predictor-based methodology we have used throughout the manuscript. For this reason, we have entirely removed this part of the fragmentation section, along with the 6 figures in the supplementary material. This section has instead been replaced by a paragraph that describes how fragmentation technique/energy impacts the MS2 spectra, and how this information is used in ML.

-----------

The authors highlight that internal fragmentation of peptides could be used as a valuable resource to implement in machine learning. There has already been some success using these fragmentation patterns for sequence identification within both top-down and bottom up proteomic searches that the authors should consider discussing. However,

-----------

As we removed the majority of this section, most of this comment is no longer relevant. We did, however, discuss and add references for the usage of fragmentation patterns in ML in proteomics.

-----------
