# Supplementary material for: Variability analysis of LC-MS experimental factors and their impact on machine learning
Source: Gigascience. 2023 Nov 20;12:giad096. doi: 10.1093/gigascience/giad096 (PMC10659119; doi:10.1093/gigascience/giad096)
Supplement: giad096_Response_to_Reviewer_Comments_Revision_1 [file giad096_response_to_reviewer_comments_revision_1.pdf]

Author's Response To Reviewer Comments

We again want to thank both reviewers for their excellent feedback.

Reviewer #1:
I am glad to see that the authors have revised the manuscript based on the reviewer's comments and improved its quality. However, the responses to some comments did not fully convince me. I suggest the authors further revise or explain the following issues.

1. I agree the rationality of ΔRT as a performance measure, but does not agree with the author's viewpoint of 'However, as the model performance indicates metric variance, and there are no changes to the conclusions drawn from the model performance'. I suggest the authors truthfully provide other classic machine learning performance metrics on the test dataset and analyze the differences.

—--------- Answer —--------------------
We are slightly surprised by the reviewer’s point. It seems that the reviewer has mis-read our original response, as we wrote 'However, as the model performance indicates metric invariance , and there are no changes to the conclusions drawn from the model performance', and not ‘metric variance’ as the reviewer comment suggests. However, this also indicates that we failed to explain the methodology properly. We also apologize for the apparent lack of visibility of the additional metrics. We already added a supplementary file with results for each model with 2 additional industry-standard metrics (MSE and MAE) and concluded that all metrics show near-identical ratio trends between training, validation and test results. To address this in the manuscript, we have added the following section paragraph to the Results and Discussions section:
“All models were measured with several metrics, namely RTΔ, mean squared error (MSE), and mean absolute error (MAE), and the performance for all metrics can be found in the supplementary evaluation_metric_report.pdf file. However, when analyzing the model performances, we observe near-identical metric ratios between training, validation, and testing across various evaluation metrics. This suggests that the outcomes remain invariant across metrics and do not alter the conclusions drawn from the models’ performance. For this reason, we have decided only to report the RTΔ values and refer to the evaluation metric report for further metric comparison”

2. In order to avoid randomness caused by single data partitioning (training and testing data partitioning), multiple random data partitioning strategie (100 or 50 times) is usually adopted to evaluate the performance of learners using multiple average performance measures and variance. It is recommended that the authors consider this issue.

—--------- Answer —--------------------
While acknowledging the importance of such a strategy, we are not able to run such computationally expensive calculations. In fact, conducting n randomized data partitions akin to cross-validation is simply not feasible for almost any deep-learning project, as training models for each partition are far too strenuous. Our training was performed with a Tesla V100 GPU and took upwards of 24 hours for the longest models, training n separate models is not computationally feasible, especially this late in the process. The most common methodology in deep learning is single training, validation, and test datasets, the exact practice we had for all our models. We even went further by stratifying the test datasets based on PRIDE projects to reduce bias in test results.
We believe that a few (>10) randomized training, validation and test partitions might have been warranted if we were to showcase a new model architecture or data processing pipeline, to highlight the ability of such an application. However, we are using current proven methodologies to showcase dataset pitfalls in the field, and adding n randomized partitions would not provide a valuable enough insight into these matters to warrant the late and substantial amount of computational needs.

3. The structure and references of the papers I have seen that have been officially published in GigaScience are very different from the manuscript (the author claimed to have organized and written

according to the requirements). I am not sure if it was my mistake or the authors' mistake. I suggest the authors confirm the issue again and improve writting.

—--------- Answer —-------------------
We have re-ordered the section following the editor's comments in the received email. We also went over the manuscript and fixed minor grammatical and writing errors. In the editors' email, the editor also mentioned registering the tool in SciCrunch.org and bio.tools. However, in this manuscript, we do not provide a new software tool targeting users but a specialized source code for this one project, which is provided on GitHub. For this reason, we do not believe that registering this source code as a separate tool is necessary, but want to iterate that we will add it if the editor insists on this.

Reviewer #2:
The changes and additions to the discussion for the paper address the key points, and have addressed some of the limitations imposed by the availability and ability to extract certain data elements particularly around sample preparation and LC settings. I thinkthis strengthens their manuscript, and provides a more wholistic discussion of factor in the experimental setup

—--------- Answer —-------------------
We completely agree that these new sections were needed to strengthen the manuscript, and we thank the reviewer for bringing these up.
