# Supplementary material for: Variability analysis of LC-MS experimental factors and their impact on machine learning
Source: Gigascience. 2023 Nov 20;12:giad096. doi: 10.1093/gigascience/giad096 (PMC10659119; doi:10.1093/gigascience/giad096)
Supplement: giad096_Response_to_Reviewer_Comments_Revision_2 [file giad096_response_to_reviewer_comments_revision_2.pdf]

Author's Response To Reviewer Comments

Reviewer: I can understand the training cost of deep learning, and in fact, we have also conducted similar training. But from a statistical or implementable perspective, I still believe that randomized experiments are essential. Even if only 5-10 repeated partitioning experiments are conducted, it can still improve sufficient validation of the method's effectiveness.
Answer: While we agree with the reviewer's statement that such partitioning can improve the validation, we do not see sufficient reason to run such time-consuming and computational intensive training of the model. We added the following sentences about this in the Conclusions section of the manuscript:
"An alternative approach would have involved performing multiple randomized data splits for each model and averaging the results to provide a more comprehensive assessment of model performance. However, we chose not to pursue this option due to their computational costs. "

We also registered our code in both bio.tools and SciCrunch, and added the identifiers to the manuscript.
