# Supplementary material for: Variability analysis of LC-MS experimental factors and their impact on machine learning
Source: Gigascience. 2023 Nov 20;12:giad096. doi: 10.1093/gigascience/giad096 (PMC10659119; doi:10.1093/gigascience/giad096)
Supplement: giad096_Reviewer_1_Report_Original_Submission — Juntao Li -- 5/25/2023 [file giad096_reviewer_1_report_original_submission.pdf]

Reviewer Report

Title: Variability Analysis of LC-MS Experimental Factors and Their Impact on Machine Learning

Version: Original Submission **Date:** 5/25/2023

Reviewer name: Juntao Li

Reviewer Comments to Author:

This paper aimed to facilitate machine learning efforts in mass spectrometry data by conducting a systematic analysis of the potential sources of variance in public mass spectrometry repositories. This paper examined how these factors affect machine learning performance and performed a comprehensive transfer learning to evaluate the benefits of current best practice methods in the field for transfer learning. Although the experimental content is extensive and provides promising results, some major points need to be addressed as follows:
1.Please explain the rationality of the RT used for evaluating model performance. In addition, it is necessary to increase other evaluation metrics to provide a more powerful comparison of model performance.
2.The curves in Figures 6 and 8 should provide more explanations to help readers understand. In addition, all figures are somewhat blurry and clearer figures should be provided.
3.This paper does not provide specific implementation steps of variance. Please describe the variance analysis process in mathematical language and provide the corresponding mathematical formula.
4.There are some formatting issues: Keywords and the title 'Data Description' should only have the first letter capitalized. On pages 6, 17, and 18, the font size of the article is inconsistent.
5.There are some grammar issues: On pages 6 and 16, dataset should be added with 's'. On page 7, lines 9-10, the tense is not unified.
6.There are significant issues with the format of references. Inconsistent capitalization of initial letters in literature titles, such as [1] and [5]; Some literature lacks page numbers, such as [6] and [18]. Please re-organize the references according to the format required by the journal.

Methods

Are the methods appropriate to the aims of the study, are they well described, and are necessary controls included? Choose an item.

Conclusions

Are the conclusions adequately supported by the data shown? Choose an item.

Reporting Standards

Does the manuscript adhere to the journal's guidelines on minimum standards of reporting? Choose an item.

Choose an item.

Statistics

Are you able to assess all statistics in the manuscript, including the appropriateness of statistical tests used? Choose an item.

Quality of Written English

Please indicate the quality of language in the manuscript: Choose an item.

Declaration of Competing Interests

Please complete a declaration of competing interests, considering the following questions:

- Have you in the past five years received reimbursements, fees, funding, or salary from an organisation that may in any way gain or lose financially from the publication of this manuscript, either now or in the future?
- Do you hold any stocks or shares in an organisation that may in any way gain or lose financially from the publication of this manuscript, either now or in the future?
- Do you hold or are you currently applying for any patents relating to the content of the manuscript?
- Have you received reimbursements, fees, funding, or salary from an organization that holds or has applied for patents relating to the content of the manuscript?
- Do you have any other financial competing interests?
- Do you have any non-financial competing interests in relation to this paper?

If you can answer no to all of the above, write 'I declare that I have no competing interests' below. If your reply is yes to any, please give details below.

I declare that I have no competing interests.

I agree to the open peer review policy of the journal. I understand that my name will be included on my report to the authors and, if the manuscript is accepted for publication, my named report including any attachments I upload will be posted on the website along with the authors' responses. I agree for my report to be made available under an Open Access Creative Commons CC-BY license (http://creativecommons.org/licenses/by/4.0/). I understand that any comments which I do not wish to be included in my named report can be included as confidential comments to the editors, which will not be published.

Choose an item.

To further support our reviewers, we have joined with Publons, where you can gain additional credit to further highlight your hard work (see: https://publons.com/journal/530/gigascience). On publication of this paper, your review will be automatically added to Publons, you can then choose whether or not to claim your Publons credit. I understand this statement.

Yes Choose an item.
