# Supplementary material for: Variability analysis of LC-MS experimental factors and their impact on machine learning
Source: Gigascience. 2023 Nov 20;12:giad096. doi: 10.1093/gigascience/giad096 (PMC10659119; doi:10.1093/gigascience/giad096)
Supplement: giad096_Reviewer_1_Report_Revision_1 — Juntao Li -- 8/28/2023 [file giad096_reviewer_1_report_revision_1.pdf]

**Reviewer Report**

**Title: Variability Analysis of LC-MS Experimental Factors and Their Impact on Machine Learning**

**Version: Revision 1** **Date:** 8/28/2023

**Reviewer name: Juntao Li**

**Reviewer Comments to Author:**

I am glad to see that the authors have revised the manuscript based on the reviewer's comments and improved its quality. However, the responses to some comments did not fully convince me. I suggest the authors further revise or explain the following issues.
1. I agree the rationality of Î"RT as a performance measure, but does not agree with the author's viewpoint of 'However, as the model performance indicates metric variance, and there are no changes to the conclusions drawn from the model performance'. I suggest the authors truthfully provide other classic machine learning performance metrics on the test dataset and analyze the differences.
2. In order to avoid randomness caused by single data partitioning (training and testing data partitioning), multiple random data partitioning strategie (100 or 50 times) is usually adopted to evaluate the performance of learners using multiple average performance measures and variance. It is recommended that the authors consider this issue.
3. The structure and references of the papers that I have seen that have been officially published in GigaScience are very different from the manuscript (the author has claimed to have organized and written according to the requirements). I am not sure if it was my mistake or the authors' mistake. I suggest the authors confirm the issue again and improve writting.

**Methods**

Are the methods appropriate to the aims of the study, are they well described, and are necessary controls included? Choose an item.

**Conclusions**

Are the conclusions adequately supported by the data shown? Choose an item.

**Reporting Standards**

Does the manuscript adhere to the journal's guidelines on minimum standards of reporting? Choose an item.

Choose an item.

**Statistics**

Are you able to assess all statistics in the manuscript, including the appropriateness of statistical tests used? Choose an item.

**Quality of Written English**

Please indicate the quality of language in the manuscript: Choose an item.

**Declaration of Competing Interests**

Please complete a declaration of competing interests, considering the following questions:

- Have you in the past five years received reimbursements, fees, funding, or salary from an organisation that may in any way gain or lose financially from the publication of this manuscript, either now or in the future?
- Do you hold any stocks or shares in an organisation that may in any way gain or lose financially from the publication of this manuscript, either now or in the future?
- Do you hold or are you currently applying for any patents relating to the content of the manuscript?
- Have you received reimbursements, fees, funding, or salary from an organization that holds or has applied for patents relating to the content of the manuscript?
- Do you have any other financial competing interests?
- Do you have any non-financial competing interests in relation to this paper?

If you can answer no to all of the above, write 'I declare that I have no competing interests' below. If your reply is yes to any, please give details below.

I declare that I have no competing interests.

I agree to the open peer review policy of the journal. I understand that my name will be included on my report to the authors and, if the manuscript is accepted for publication, my named report including any attachments I upload will be posted on the website along with the authors' responses. I agree for my report to be made available under an Open Access Creative Commons CC-BY license (http://creativecommons.org/licenses/by/4.0/). I understand that any comments which I do not wish to be included in my named report can be included as confidential comments to the editors, which will not be published.

Choose an item.

To further support our reviewers, we have joined with Publons, where you can gain additional credit to further highlight your hard work (see: https://publons.com/journal/530/gigascience). On publication of this paper, your review will be automatically added to Publons, you can then choose whether or not to claim your Publons credit. I understand this statement.

Yes Choose an item.
