# Supplementary material for: Variability analysis of LC-MS experimental factors and their impact on machine learning
Source: Gigascience. 2023 Nov 20;12:giad096. doi: 10.1093/gigascience/giad096 (PMC10659119; doi:10.1093/gigascience/giad096)
Supplement: giad096_Reviewer_2_Report_Original_Submission — Luke Carroll -- 6/19/2023 [file giad096_reviewer_2_report_original_submission.pdf]

Reviewer Report

Title: Variability Analysis of LC-MS Experimental Factors and Their Impact on Machine Learning

Version: Original Submission **Date:** 6/19/2023

Reviewer name: Luke Carroll

Reviewer Comments to Author:

The paper applies machine learning to publicly available proteomics data sets and assesses the ability to transfer learning algorithms between projects. The primary aim of these algorithms appears to be an attempt to increase consistency of retention time prediction for data-dependent acquisition data sets, however this is not explicitly stated within the text. The application of machine learning to derive insight from previous performed proteomics experienced is a worthwhile exercise.

1. The authors report Î"RT to determine fitting for their models. It would be interesting to see whether the models had other metrics used to assess fitting, or could be used to increase number of identifications within sample sets, and whether this was successful. ALternatively, was there any conclusions able to be drawn about peptide structure and RT determination from these models?
2. Project specific libraries are well known to improve results compared with publicly available databases, and the discussion on this point should be developed further through comparison of this work with other papers - particularly with advances in machine learning and neural networks in the data independent analysis field.
3. Comparison of Q-Exactiv models vs Orbitraps appears to be somewhat redundant, and possible a result of poor meta-data as Q-Exactiv instruments are orbitrap mass spectrometers. A more interesting comparison to make here would be between orbitrap and TOF instruments, though as the datasets have all been processed through MaxQuant, it is likely the vast majority were acquired on orbitrap instruments.
4. The paper uses Î"RT as the readout for all models tested, however the only chromatography variable considered in testing the models is gradient length. However, chromatography is also dependent on column chemistry, column dimensions, composition of buffer, use of traps, temperature etc. These are also likely to be contributing the variance observed between the PT datasets where these variables will be consistent and publicly available datasets. These factors are also likely to play a role in higher uncertainty for early and late eluting peptides where these factors are likely to vary most between sample sets. The metadata may not be available to use to compare within the data sets selected, so the authors should at minimum make discussion around these points.
5. Sample preparation is likely to have similar effects, and as the PT datasets are generated synthetically using ideal peptides, publicly available datasets will be generated from complex sample mixtures, and have increased variance due to inefficiencies of digestion, sample clean up and matrix effects. Previous studies on variance have also described sample preparation as the highest cause of variance. This needs further discussion
6. While the isolation windows of the m/z will lead to unobserved space, search engines setting will also apply here. From the text, it appears that the only spectra that were considered were those already

identified in a search program (due to having Andromeda cut-off scores always apply). Typical setting for a database search will have a cut off of peptide sequences of at least 7 residues, making peptide masses appearing lower than 350 m/z unlikely. There is also significant amount of noise below 350 m/z and this also likely contributes to poorer fitting.

7. The authors identify differences in MSMS spectral features, however, most of these points are well known in the field. The authors should develop the discussion on the causes of the differences in fragmentation, as CID low mass drop off is expected, and the change in profile is expected with increasing activation energies. A more developed analysis could exclude precursor masses from these plots and focus solely on fragment ions generated.

8. The authors highlight that internal fragmentation of peptides could be used as a valuable resource to implement in machine learning. There has already been some success using these fragmentation patterns for sequence identification within both top-down and bottom up proteomic searches that the authors should consider discussing. However, these data do not appear to be incorporated into the machine learning models in this paper - or at least seem not to play a significant role in prediction, and this section appears to be a bit out of place.

Methods

Are the methods appropriate to the aims of the study, are they well described, and are necessary controls included? Choose an item.

Conclusions

Are the conclusions adequately supported by the data shown? Choose an item.

Reporting Standards

Does the manuscript adhere to the journal's guidelines on minimum standards of reporting? Choose an item.

Choose an item.

Statistics

Are you able to assess all statistics in the manuscript, including the appropriateness of statistical tests used? Choose an item.

Quality of Written English

Please indicate the quality of language in the manuscript: Choose an item.

Declaration of Competing Interests

Please complete a declaration of competing interests, considering the following questions:

- Have you in the past five years received reimbursements, fees, funding, or salary from an organisation that may in any way gain or lose financially from the publication of this manuscript, either now or in the future?
- Do you hold any stocks or shares in an organisation that may in any way gain or lose financially from the publication of this manuscript, either now or in the future?
- Do you hold or are you currently applying for any patents relating to the content of the manuscript?
- Have you received reimbursements, fees, funding, or salary from an organization that holds or has applied for patents relating to the content of the manuscript?
- Do you have any other financial competing interests?
- Do you have any non-financial competing interests in relation to this paper?

If you can answer no to all of the above, write 'I declare that I have no competing interests' below. If your reply is yes to any, please give details below.

I declare that I have no competing interests

I agree to the open peer review policy of the journal. I understand that my name will be included on my report to the authors and, if the manuscript is accepted for publication, my named report including any attachments I upload will be posted on the website along with the authors' responses. I agree for my report to be made available under an Open Access Creative Commons CC-BY license (http://creativecommons.org/licenses/by/4.0/). I understand that any comments which I do not wish to be included in my named report can be included as confidential comments to the editors, which will not be published.

Choose an item.

To further support our reviewers, we have joined with Publons, where you can gain additional credit to further highlight your hard work (see: https://publons.com/journal/530/gigascience). On publication of this paper, your review will be automatically added to Publons, you can then choose whether or not to claim your Publons credit. I understand this statement.

Yes Choose an item.
